# Synthesis and *In Silico* Docking of New Pyrazolo[*4,3-e*]pyrido[*1,2-a*]pyrimidine-based Cytotoxic Agents

**DOI:** 10.3390/ijms221910258

**Published:** 2021-09-23

**Authors:** Mabrouk Horchani, Niels V. Heise, Sophie Hoenke, René Csuk, Abdel Halim Harrath, Hichem Ben Jannet, Anis Romdhane

**Affiliations:** 1 Laboratory of Heterocyclic Chemistry, Natural Products and Reactivity, Medicinal Chemistry and Natural Products (LR11ES39), Faculty of Sciences Monastir, University of Monastir, Monastir 5000, Tunisia; horchani.mabrouk@gmail.com (M.H.); anis_romdhane@yahoo.fr (A.R.); 2 Organic Chemistry, Martin-Luther-University Halle-Wittenberg, Kurt-Mothes-Str. 2, D-06120 Halle (Saale), Germany; niels.heise@student.uni-halle.de (N.V.H.); sophie.hoenke@chemie.uni-halle.de (S.H.); 3 College of Science, Department of Zoology, King Saud University, Riyad 11451, Saudi Arabia; hharrath@ksu.edu.sa

**Keywords:** pyrazolo-pyrido-pyrimidines, cytotoxicity, tumor cell lines, SAR, in silico docking

## Abstract

To explore a new set of anticancer agents, a novel series of pyrazolo[4,3-*e*]pyrido[1,2-*a*]pyrimidine derivativeshave been designed and synthesized viacyclocondensation reactions of pyrazolo-enaminone with a series of arylidenemalononitriles; compound **5** was obtained from 5-amino-4-cyanopyrazole. The structures of the target compounds were investigated by spectral techniques and elemental analysis (IR, UV–Vis, ^1^H NMR, ^13^C NMR and ESI-MS). All compounds were evaluated for their in vitro cytotoxicity employing a panel of different human tumor cell lines, A375, HT29, MCF7, A2780, FaDu as well as non-malignant NIH 3T3 and HEK293 cells. It has been found that the pyrazolo-pyrido-pyrimidine analog bearing a 4-Br-phenyl moiety was the most active toward many cell lines with EC_50_ values ranging between 9.1 and 13.5 µM. Moreover, in silico docking studies of the latter with six anticancer drug targets, i.e., DHFR, VEGFR2, HER-2/neu, hCA-IX, CDK6 and LOX5, were also performed, in order to gain some insights into their putative mode of binding interaction and to estimate the free binding energy of this bioactive molecule.

## 1. Introduction

At present, cancer still constitutes a tremendous frightful disease for numerous patients worldwide. This disease is regarded as one of the foremost causes of mortality, thus endangering the health and life of humans. Furthermore, cancer cases surpassed 14 million persons in 2012, and it is expected to affect 22 million persons in 2030 [1]. Regardless of the state of development and economic prosperity in all countries worldwide, the number of persons suffering from malignant melanoma, colon, breast, human ovarian carcinoma or pharynx carcinoma increased during the last decade. Under these circumstances, besides surgery and radiotherapy, chemotherapy is the most currently used treatment to cure the various types of cancers [2]. Nowadays, chemotherapy applying a set of drugs acting by different mechanisms is one of the most promising techniques being applied to treat cancer.

Thus, in spite of all progress to treat and cure cancer patients, there is still need to search for and to develop novel anticancer agents holding superior efficacy but giving rise to minimal side effects. Therefore, the identification of new chemical entities that are more reliable and efficient remains a major challenge for medicinal chemists. As a consequence, the synthesis of small molecules still represents a potent and effective strategy to supply novel chemical entities for cancer therapy.

**Figure 1:**
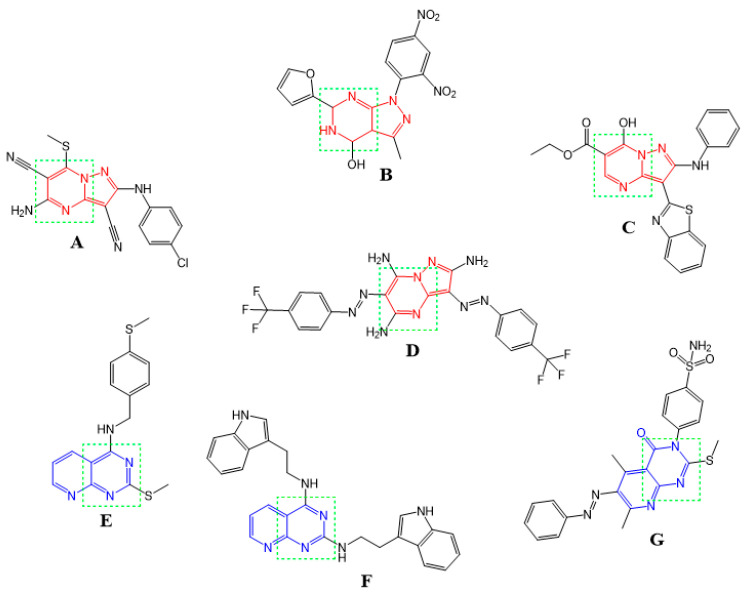
Previously reported cytotoxic compounds: pyrazole-fused pyrimidines (**A**–**D**) and pyridine-fused pyrimidines (**E**–**G**).

In this context, heterocyclic compounds, especially those holding more than one non-carbon ring atom have played a key role in the field of medicinal chemistry [3,4]. Furthermore, the previously reported results from literature reveal that the combination of two or more bioactive heterocyclic pharmacophores into the same molecule appears to be an effective tool for designing new chemical entities that have improved activity. Thereby, pyrazole-fused pyrimidines constitute a promising versatile class of heterocyclic scaffolds having always attracted much interest from chemists owing to their outstanding potential, such as antitumor [5], anti-inflammatory [6], anticancer and anti-5-lipoxygenase [7], antibacterial [8], antitubercular [9] and especially cytotoxic activities (Figure 1A–D) [10,11,12,13].

Pyrido-pyrimidines have, for a long time, attracted the interests of both biological and synthetic researchers alike due to their various biological therapeutic properties such as anticonvulsant [14], antitumor [15], anti-proliferative [16], antifungal [17], adenosine kinase inhibition [18] and cytotoxic activities (Figure 1E–G) [19,20].

**Figure 2:**
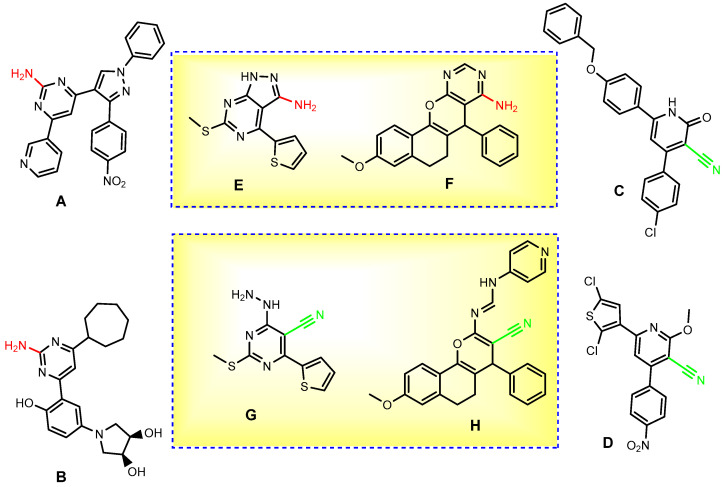
Previously reported cytotoxic compounds tethered with amino (**A**,**B**,**E**,**F**) or cyano (**C**,**D**,**G**,**H**) groups.

A review of the literature has shown in several cases that amino and cyano functions can be involved in some interesting interactions with target enzymes. Indeed, various constructed heterocyclic compounds gained much attention owing to their cytotoxic and anticancer activities especially in cases wherethe NH_2_ group shows conjugation when it is tethered with a pyrimidine (Figure 2A,B) [21,22] or the CN group linked in a conjugate way with a pyridine (Figure 2C,D) [23,24].

In addition, a review of the literature revealed that several strongly cytotoxic chemical scaffolds contain in their structures amine (Figure 2E,F) or cyano (Figure 2G,H) groups directly linked to a heterocycle [25,26]. This particular structural linkage gave the kick-off to several research teams to design and synthesize amino-pyrimidine and cyano-pyridine scaffolds due to this notable anticancer activity against a wide range of cell lines.

Inspired by the above findings, and in continuation of our previous work on the synthesis of novel fused-pyrimidine scaffolds [27,28,29], we aimed to design and synthesize new pyrazolo[4,3-*e*]pyrido[1,2-*a*]pyrimidine derivatives. Hereby, we report for the first time the cytotoxic activity of these newly synthesized compounds toward five human tumor cell lines, A375, HT29, MCF7, A2780, FaDu as well as non-malignant NIH 3T3 and HEK293 cells. Moreover, their structure–activity relationship (SAR) was investigated.

## 2. Results and Discussion

### 2.1. Synthesis

**Scheme 1:**
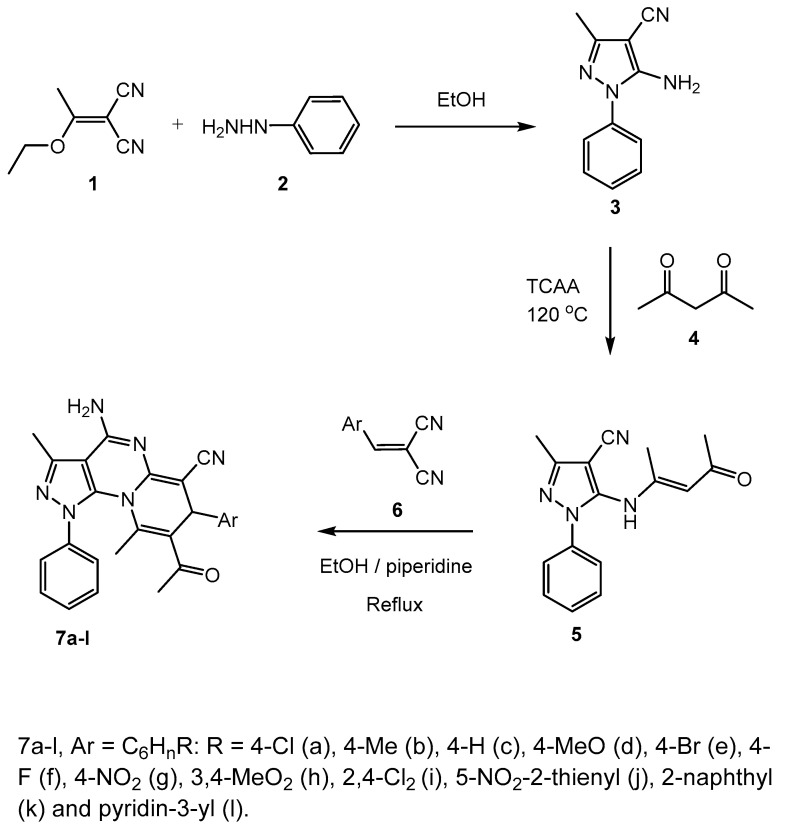
Synthetic pathway for the synthesis of pyrazolo-pyrido-pyrimidine derivatives **7a**–**l**.

The multistep synthesis of target compounds **7a**–**l** (Scheme 1) started from the bi-nucleophilic precursor **3**; the latter was prepared according to a previously published literature procedure [30] starting from 2-(1-ethoxyethylidene)malononitrile(**1**) and phenylhydrazine (**2**). Compound **5**, an enaminone holding a pyrazole moiety, was obtained from the nucleophilic addition reaction of 5-amino-3-methyl-1-phenyl-1*H*-pyrazole-4-carbonitrile (**3**) and acetylacetone (2,4-pentanedione, **4**) in the presence of a catalytic amount of trichloroacetic acid (TCAA) acting as a catalyst under solvent-free conditions. In the next step, cyclo-condensation reactions of **5** and a series of arylidenemalononitriles **6** led to the title polyheterocyclic scaffolds **7a**–**l** (Scheme 1).

**Scheme 2:**
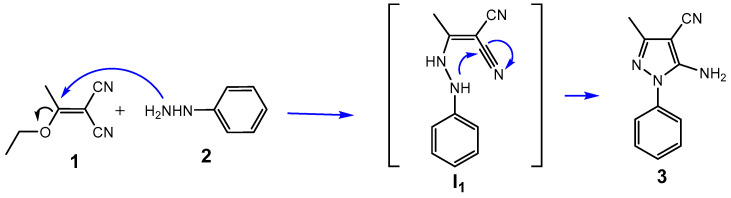
Plausible mechanistic pathway for the synthesis of target compound **3**.

In detail, the use of aminopyrazoles as heterocyclizing agents has received considerable attention in recent years. As a result, these molecules have proven to be the building blocks of choice in the preparation of several heterocyclic compounds, they in fact exhibit high reactivity due to the presence of a primary amine function and of a nitrile function in the alpha position. These two functions are capable of reacting as a nucleophilic and electrophilic agent, respectively, and thus subsequently undergo the cyclization reaction. This is why we chose to synthesize this type of amino-cyanopyrazole **3**. Mechanistically, the reaction begins with the attack by the free doublet of the primary amine of phenylhydrazine on the ethylenic quaternary carbon of the ether function in the ethoxyalkylidene **1**, releasing an ethanol molecule, then a second nucleophilic attack on the nitrile function by the secondary amine function of the phenylhydrazine leads to the expected pyrazole compound **3** (Scheme 2).

**Scheme 3:**
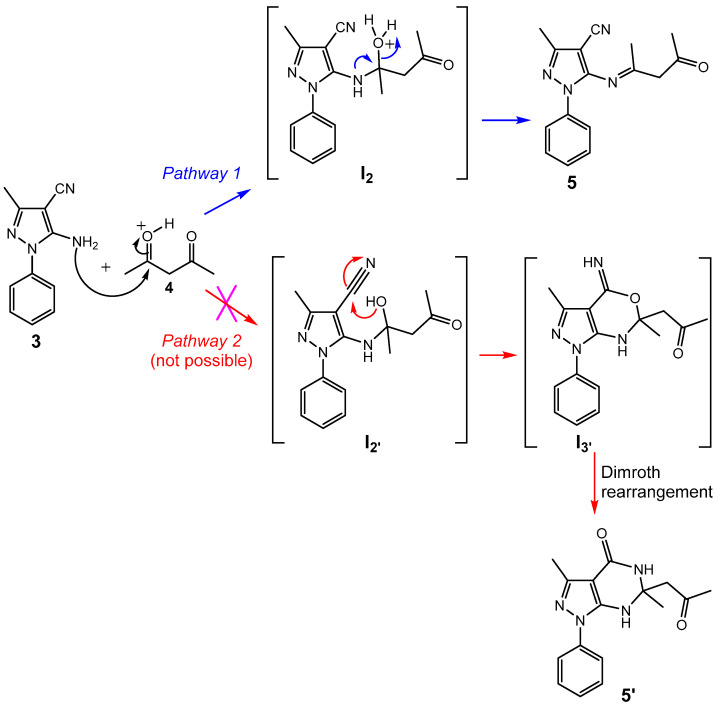
Plausible mechanistic pathway for the synthesis of compound **5**.

The use of TCAA (pka = 0.77) facilitates the formation of an electrophilic site (C=O) of the acetylacetone **4**, and therefore makes it easy to attack the primary amine’s free doublet of the starting building block **3**,which gives a non-isolable intermediate **I_2_**. The crucial role of TCAA can be seen again through the facilitation of the dehydration made by a second nucleophilic attack of the free doublet of the same amine function on the same electrophilic site, which gives the isolable intermediary **5** (pathway 1). These conditions imply the failure to obtain compound **5′** (pathway 2) (Scheme 3).

**Scheme 4:**
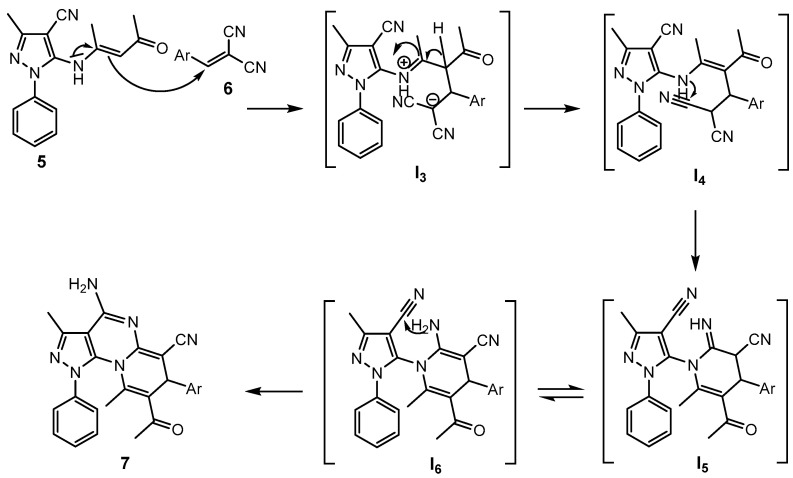
Putative mechanistic pathway for the synthesis of target compounds **7a**–**l**.

A putative mechanism (Scheme 4) for the formation of **7a**–**l** has been depicted in Scheme 2. Thereby, the reaction sequence starts with a nucleophilic attack onto the double bond of the enaminone; this Michael addition forms intermediate **I_3_**, which subsequently undergoes a proton transfer from one carbon to another one followed by an intramolecular cyclization to afford the non-isolable intermediate **I_5_**. Upon tautomerization, a primary amine is formed, which attacks the nitrile function of the pyrazole moiety leading finally to target compound **7**.

### 2.2. Biological Evaluation

The synthesized compounds were evaluated for their cytotoxic activity by a photometric rhodamine B assay (SRB) on different human cancer cell lines, malignant melanoma (A375), colon (HT29), breast (MCF-7), human ovarian carcinoma (A2780) and pharynx carcinoma (FaDu); for comparison, non-malignant mouse embryonic fibroblasts (NIH 3T3) and human embryonic kidney cells (HEK293) were included; doxorubicin (DX) was used as a positive control [31,32]. The results are summarized in Table 1.The EC_50_ values in μM from SRB assays were determined after 72 h of treatment, and the values are averaged from three independent experiments performed each in triplicate, confidence interval, CI = 95%; mean ± standard mean error).

The intermediates **3** and **5** were found to be non-cytotoxic (EC_50_ > 30 µM) against all the five cancer cell lines. However, many of the target compounds (**7a**–**h**) showed noteworthy cytotoxic effects for all tested human tumor cell lines.

**Figure 3:**
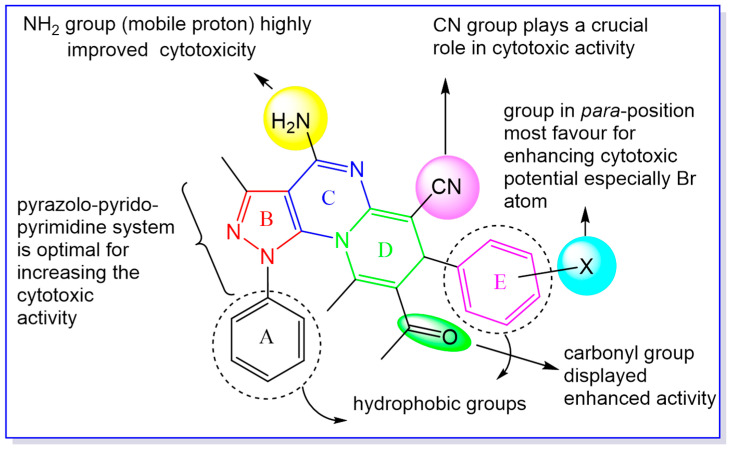
The structure–activity relationship (SAR) of designed scaffolds cytotoxic agent **7**.

Our target compound **7** holds five cyclic rings, A, B, C, D and E (Figure 3). The values of EC_50_ differ by the group linked to cycle E. Interestingly, the highest activity in A375 cancer cell was determined for compound **7e** with a bromine substituent attached to the aryl moiety E in the *para* position. The next molecule in this series was the methyl-substituted compound **7b**, also exhibiting good cytotoxicity (EC_50_ = 12.9 ± 1.6 µM), while nitro-substituted compound **7g** was less cytotoxic (EC_50_ = 16.2 ± 2.0 µM), followed by **7f**, **7a** and **7c**, respectively. On the other hand, the topmost activity in colon cancer cells (HT29) was again determined for **7e** holding an EC_50_ value of 13.3 ± 1.8 µM, followed by **7b**, **7f** (Ar = 4-F-Ph) and **7g** with EC_50_ values of 17.5 ± 1.8, 24.7 ± 4.4, 25.9 ± 3.4 µM, respectively. Toward MCF-7, it is apparent that the same derivative **7e** exhibited the highest activity with an EC_50_ value of 9.2 ± 0.7µM, and compounds **7b** and **7g** (EC_50_= 12.2 ± 1.3 and 15.7 ± 2.2 µM, respectively) also exhibited good activities.

The highest cytotoxicity for A2780 cancer cell lines was found again for compound **7e** (EC_50_ = 9.1 ± 1.6 µM). In this series, compounds **7g** and **7b** (EC_50_= 14.5 ± 2.2 and 14.6 ± 2.2 µM, respectively) showed noteworthy activity as compared to other analogs, and their EC_50_ values ranged between 18.0 ± 3.0 and 27.5 ± 8.1µM. Unfortunately, compound **7e** also showed significant cytotoxic effects for the non-malignant cell lines. Our compounds are significantly less cytotoxic than “gold standard”doxorubicin (DX). This may be due to the fact that DX acts in two ways in cancer cells: on the one hand, DX produces free radicals and thus damages cellular membranes, and on the other hand, DX interacts with DNA and disrupts topoisomerase-II-mediated DNA repair. Conjugation of our compounds with functional groups that have a particularly high affinity for cellular membranes could lead to more cytotoxic compounds. This would also represent a possibility to achieve higher tumor-cell/non-tumor cell selectivity.

Since the SRB assays showed **7e** as the most active compound, molecular docking was performed to establish some structure–activity relationships (SARs). The increase in the activity of **7e** might be due to the presence of an electron-withdrawing group attached to the E ring.

**Figure 4:**
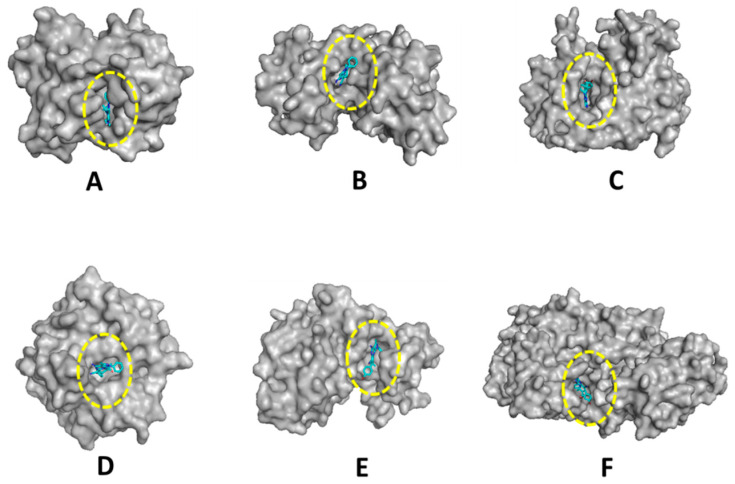
The surface representation of enzymes (**A**) DHFR, (**B**) VEGFR2, (**C**) HER-2, (**D**) hCA-IX, (**E**) CDK6 and (**F**) LOX5 in gray, and the sticks represent (cyan) the docked compound **7e**.

Today, researchers increasingly use the in silico techniques and methods, including the molecular docking, to predict potential interactions of tested compounds for their cytotoxic activity with the key targets in cancer cells. Hereby, literature has shown that six key proteins (MCL-1, CDK6, HER-2/neu, LOX5, USP7 and DHFR) [33] or (DHFR, VEGFR2, HER-2/neu, hCA-IX, CDK6 and LOX5) [32] were used in the in silico docking studies of the most potent compounds in order to investigate their predicted anticancer potential. In this study, to develop a new generation of more potent multitargeted anticancer agents and motivated by previous research on that topic [28,32,34], we took advantage of the reported structure–activity relationships (SARs) of pyrazolo-pyrido-pyrimidine analogs. Thus, in order to assess the ability of the top-ranked active compounds to inhibit six anti-cancer drug targets (DHFR, VEGFR2, HER-2/neu, hCA-IX, CDK6 and LOX5), we carried out docking study for complexes consisting of compound **7e** and the target proteins. This ligand fits very well in the active site binding cavity of all target enzymes (Figure 4).

**Figure 5:**
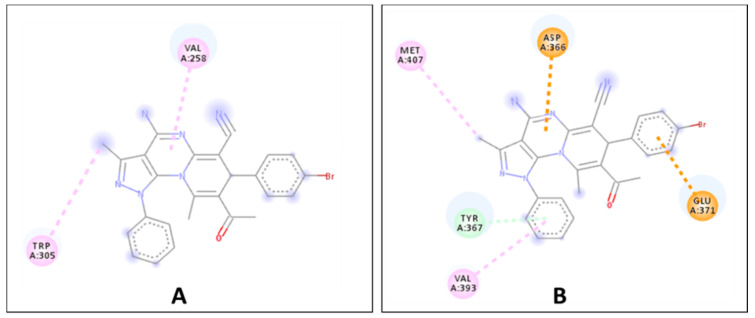
Docking pose (2D binding model) of the most active cytotoxic agent, **7e**, in the active site of MCL-1 (PDB: 3MK8) (**A**) and USP7 (PDB: 4M5W) (**B**).

The choice of these six proteins among the eight proposed above was based on the importance of the interactions shown by the docked compound with the residues forming the active site of each target enzyme, while, toward MCL-1 and USP7 enzymes, the ligand **7e** did not show interesting interactions, and especially the absence of hydrogen bonds (Figure 5).

The obtained results showed excellent interaction with all the above targets as compared to their respective co-crystallized ligands (Table 2).

**Figure 6:**
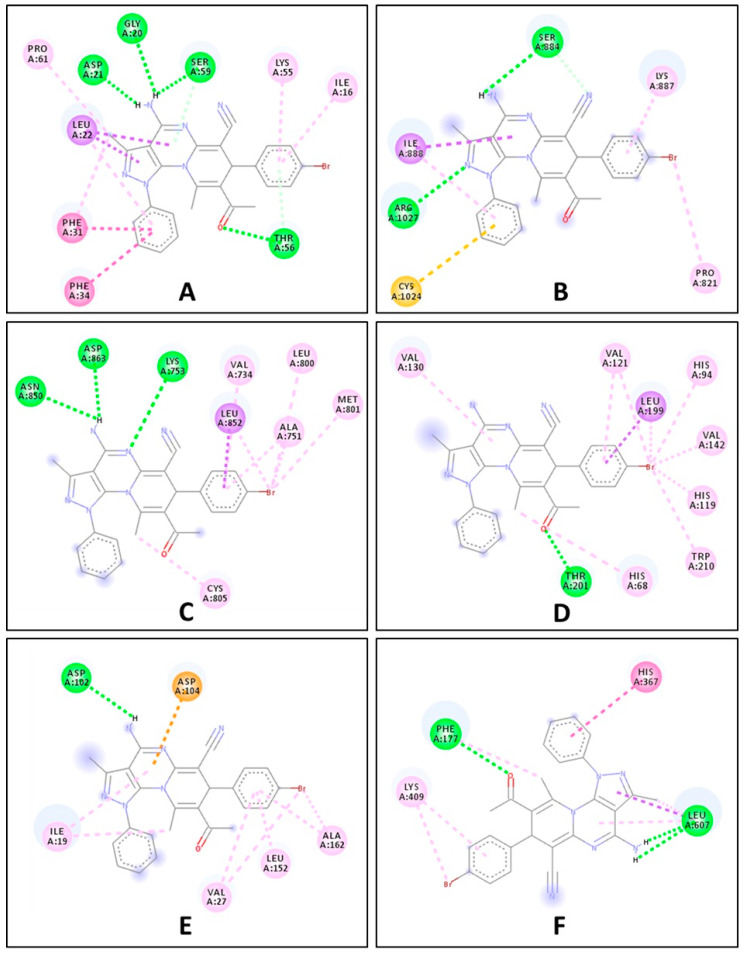
Docking pose (2D binding model) of the most active cytotoxic agent, **7e**, in the active site of DHFR (PDB: 5HQY) (**A**), VEGFR2 (PDB: 5EW3) (**B**), HER-2 (PDB: 3RCD) (**C**), hCA-IX (PDB: 5FL4) (**D**), CDK6 (PDB: 3NUP) (**E**) and LOX5 (PDB: 3V99) (**F**).

Considering interactions with all the targets under observation, ligand **7e** fits well inside the pocket; it displayed crucial hydrogen bonds by its (CO) and (NH_2_) groups; thereby in the case of the most favorable complex of compound **7e** with DHFR, four H-bonds were formed: three between the amine function and GLY20, ASP21 and SER59 and one through the carbonyl group with THR56. The fused heterocyclic system seems to play a significant binding role through the appearance of a pi–sigma interaction with LEU22. In addition, the phenyl groups exhibit many hydrophobic interactions (Figure 6A). Moreover, toward active site amino acids of VEGFR2 (Figure 6B) **7e** is involved in two H-bonds with SER884 and ARG1027, a pi–sigma interaction with ILE888, a pi–sulfur interaction with CYS1024, as well as alkyl with PRO821 and pi–alkyl with LYS 887 and ILE888. Furthermore, in the case of HER-2/neu, compound **7e** forms some substantial H-bonds, i.e., two through its amine function and one by its carbonyl group, a pi–sigma interaction with LEU852 besides many hydrophobic interactions with the amino acid sequence VAL734, ALA751, LEU800, MET801 and CYS 805 (Figure 6C). In the same manner, toward active site amino acids of hCA-IX, ligand **7e** formed a conventional hydrogen bond by its carbonyl group with THR201, showed pi–sigma interaction with LEU199 and hydrophobic interactions with residues HIS68, HIS94, HIS119, VAL121, VAL130, VAL142 and TRP210 (Figure 6D). Furthermore, for the complex CDK6–**7e**, a hydrogen bond formed between (NH_2_) and ASP102, pi–anion with ASP104 and hydrophobic interactions with ILE19, VAL27, LEU152 and ALA162 (Figure 6E) were found. Finally, against LOX, **7e** demonstrated three H-bonds through its CO and NH_2_ groups with PHE177 and LEU607 and showed some hydrophobic interactions with the amino acids sequence PHE177, HIS367, LYS409 and LEU607 (Figure 6F).

## 3. Materials and Methods

### 3.1. General

A detailed description of materials and methods can be found in the Supplementary Materials file. For molecular docking, the 3D crystal structures of PDB were obtained from the RSCB protein data bank (PDB): DHFR (ID:5HQY[35]), VEGFR2 (ID:5EW3 [35,36]), HER2/neu (ID:3RCD [35,37]), hCA-IX (ID:5FL4 [35,38]), CDK6 (ID:3NUP [35,39]) and LOX5 (ID:3V99 [35,40]). All water molecules were removed, and hydrogen atoms were added before docking using Discovery Studio Visualizer D.S. (2017) (BIOVIA, San Diego, CA, USA); Gasteiger charges were also added to the system during the preparation of the receptor input file. Docking studies were performed using AutoDock 4.2 software (Scripps Research; http://autodock.scripps.edu, accessed on 7 July 2021). The structures of the compounds were drawn using ChemDraw [ver. 10.0]. The optimization of all the geometries of scaffolds was performed with ACD (3D viewer) software (http://www.filefacts.com/acd3d-viewer-freeware-info, accessed on 7 July 2021). Co-crystalized ligands in the proteins were taken as reference ligands and re-docked into the active site of proteins for energy comparison. The top-scored conformation was recorded for each compound and used for further analysis, and 2D images were captured through Discovery Studio Visualizer (2017) developed by Accelrys (BIOVIA, San Diego, CA, USA). ^13^C NMR spectra (Supplementary Materials) were recorded as APT spectra showing CH and CH_3_ groups as positive signals and CH_2_ groups and quaternary carbons as negative signals.

### 3.2. Synthesis

#### 3.2.1. 5-Amino-3-methyl-1-phenyl-1H-pyrazole-4-carbonitrile (**3**)

To an ice-cold solution of **1** (0.1 mol) in ethanol (100 mL), **2** (0.1 mol) was added, and the mixture was stirred for 3 h. The precipitate was filtered off and re-crystalized from ethanol; yield: 86%; m.p. 132 °C; R_F_ = 0.69 (SiO_2_, CHCl_3_/MeOH, 95:5); IR (ATR): ν = 3329m, 2216s, 1652m, 1596m, 1563m, 1533s, 1488m, 1444m, 1318w, 996w, 833w, 758s, 722m, 689s, 624m, 554m, 506m, 451w cm^−1^; ^1^H NMR (500 MHz, CDCl_3_): δ = 7.51–7.47 (m, 2H, 3-H, 5-H), 7.47–7.43 (m, 2H, 2-H, 6-H), 7.42–7.38 (m, 1H, 4-H), 4.67 (s, 2H, NH_2_), 2.29 (s, 3H, 11-H) ppm; ^13^C NMR (126 MHz, CDCl_3_): δ = 151.1 (C-9), 150.3 (C-7), 137.0 (C-1), 130.0 (C-3, C-5), 128.7 (C-4), 124.2 (C-2, C-6), 114.6 (C-10), 76.3 (C-8), 13.0 (C-11) ppm; MS (ESI, MeOH): *m/z* 199 (100%, [M+H]^+^), 237 (52%, [M+K]^+^); analysis calcd.for C_11_H_10_N_4_ (198.22): C 66.65, H 5.09, N 28.26; found: C 66.41, H 5.24, N 28.04.

#### 3.2.2. 3-Methyl-5-{[(1E)-1-methyl-3-oxobut-1-en-1-yl]amino}-1-phenyl-1H-pyrazole-4-carbonitrile (**5**)

A mixture of aminopyrazole **3** (7 mmol), acetylacetone**4** (7 mmol) and trichloroacetic acid (1 mmol) was stirred at 120 °C for 10 h. The precipitate was filtered off, dissolved in a minimum amount of CHCl_3_ and precipitated with petrol ether followed by chromatographic purification (SiO_2_, chloroform/ethyl acetate, 9:1): yield: 62%; m.p. 109 °C; R_F_ = 0.89 (SiO_2_, CHCl_3_/MeOH, 95:5); UV–Vis (CHCl_3_): λ_max_ (log ε) = 260 nm (3.96), 308 nm (3.96); IR (ATR): ν = 2228m, 1619s, 1574s, 1503m, 1430m, 1357w, 1274s, 1181w, 1125w, 1022w, 908w, 790w, 747s, 693s, 636w, 536w, 512s cm^−1^; ^1^H NMR (500 MHz, CDCl_3_): δ = 12.42 (s, 1H, NH), 7.53–7.27 (m, 5H, 2-H, 3-H, 4-H, 5-H, 6-H), 5.35 (s, 1H, 13-H), 2.43 (s, 3H, 11-H), 2.09 (s, 3H, 15-H), 1.94 (s, 3H, 16-H) ppm; ^13^C NMR (126 MHz, CDCl_3_): δ = 198.7 (C-14), 158.2 (C-12), 152.4 (C-7), 142.4 (C-9), 137.4 (C-1), 129.9 (C-3, C-5), 129.2 (C-4), 124.3 (C-2, C-6), 113.7 (C-10), 101.3 (C-13), 90.6 (C-8), 29.8 (C-15), 19.7 (C-16), 13.6 (C-11) ppm; MS (ESI, MeOH): *m/z* 281 (32%, [M+H]^+^), 303 (100%, [M+Na]^+^); analysis calcd.for C_16_H_16_N_4_O (280.33): C 68.55, H 5,75, N 19.99; found: C 687.37, H 5.98, N 19.75.

#### 3.2.3. General Procedure for the Synthesis of Compounds **7a**–**l**

A mixture of enaminone**5** (1 mmol) and arylidenemalononitrile**6** (1 mmol) was heated at reflux in ethanol (10 mL) in the presence of a catalytic amount of piperidine for 8 h. The solvent was removed at reduced pressure, and the crude products were purified by chromatography (SiO_2_, chloroform/ethyl acetate, 8/2).

##### 8-Acetyl-4-amino-7-(4-chlorophenyl)-3,9-dimethyl-1-phenyl-1,7-dihydropyrazolo[4,3-*e*] Pyrido[1,2-*a*]pyrimidine-6-carbonitrile (**7a**)

Yield: 78%; m.p. 167 °C; R_F_ = 0.33 (SiO_2_, CHCl_3_/MeOH, 95:5); UV–Vis (CHCl_3_): λ_max_ (log ε) = 290 nm (3.97); IR (ATR): ν = 3332w, 2185m, 1653m, 1609s, 1570m, 1540s, 1488m, 1221m, 1192w, 1090w, 1012w, 859w, 764m, 693m, 624w, 509m cm^−1^; ^1^H NMR (500 MHz, CDCl_3_): δ = 7.37–7.19 (m, 8H, 2-H, 3-H, 5-H, 6-H, 22-H, 23-H, 25-H, 26-H), 6.73 (m, 1H, 4-H), 4.97 (s, 1H, 15-H), 2.61 (s, 3H, 10-H), 2.36 (s, 3H, 19-H), 1.68 (s, 3H, 17-H) ppm; ^13^C NMR (126 MHz, CDCl_3_): δ = 197.9 (C-18), 147.6 (C-12), 146.2 (C-11), 144.1 (C-7), 143.9 (C-13), 141.4 (C-9), 140.3 (C-21), 138.0 (C-1), 133.7 (C-24), 130.1 (C-3, C-5), 129.2 (C-23, C-25), 129.2 (C-2, C-6), 128.4 (C-22, C-26), 125.5 (C-20), 124.0 (C-14), 123.1 (C-4), 100.0 (C-8), 72.2 (C-16), 40.1 (C-15), 31.0 (C-19), 14.5 (C-10) ppm; MS (ESI, MeOH): *m/z* 467 (100%, [M − H]^-^); analysis calcd.for C_26_H_21_ClN_6_O (468.94): C 66.59, H 4.51, N 17.92; found: C 66.30, H 4.72, N 17.63.

##### 8-Acetyl-4-amino-3,9-dimethyl-7-(4-methylphenyl)-1-phenyl-1,7-dihydropyrazolo[4,3-*e*] Pyrido1,2-*a*]pyrimidine-6-carbonitrile (**7b**)

Yield: 66%; m.p. 167 °C; R_F_ = 0.33 (SiO_2_, CHCl_3_/MeOH, 95:5); UV–Vis (CHCl_3_): λ_max_ (log ε) = 289 nm (4.27); IR (ATR): ν = 2185m, 1608s, 1542s, 1479m, 1449w, 1357w, 1223m, 1194w, 879w, 810w, 764m, 695m, 624w, 511m cm^−1^; ^1^H NMR (500 MHz, CDCl_3_): δ = 7.33–7.28 (m, 2H, 2-H, 6-H), 7.23–7.18 (m, 2H, 3-H, 5-H), 7.17 (s, 4H, 2-H, 3-H, 5-H, 6-H), 6.78–6.67 (m, 1H, 4-H), 4.94 (s, 1H, 15-H), 2.58 (s, 3H, 10-H), 2.38 (s, 3H, 19-H), 2.32 (s, 3H, 27-H), 1.65 (s, 3H, 17-H) ppm; ^13^C NMR (126 MHz, CDCl_3_): δ = 197.9 (C-18), 155.8 (C-12), 155.0 (C-11), 145.3 (C-7), 143.2 (C-13), 141.2 (C-9), 138.5 (C-21), 137.8 (C-1), 137.0 (C-24), 129.6 (C-3, C-5), 129.2 (C-23, C-25), 128.4 (C-2, C-6), 126.4 (C-22, C-26), 125.1 (C-20), 122.7 (C-4), 122.5 (C-14), 99.8 (C-8), 71.5 (C-16), 40.0 (C-15), 30.3 (C-19), 21.0 (C-27), 19.1 (C-17), 13.7 (C-10) ppm; MS (ESI, MeOH): *m/z*449 (100%, [M+H]^+^); analysis calcd.for C_27_H_24_N_6_O (448.52): C 72.30, H 5.39, N 18.74; found: C 72.07, H 5.51, N 18.55.

##### 8-Acetyl-4-amino-3,9-dimethyl-1,7-diphenyl-1,7-dihydropyrazolo[4,3-*e*]Pyrido[1,2-*a*]pyrimidine-6-carbonitrile (**7c**)

Yield: 62%; m.p. 198 °C; R_F_ = 0.31 (SiO_2_, CHCl_3_/MeOH, 95:5); UV–Vis (CHCl_3_): λ_max_ (log ε) = 289 nm (4.54); IR (ATR): ν = 3148w, 2181m, 1660m, 1601s, 1544s, 1480m, 1447m, 1355w, 1220m, 1197m, 878w, 764m, 722m, 694s, 627w, 511s cm^−1^; ^1^H NMR (500 MHz, CDCl_3_): δ = 7.40–7.16 (m, 9H, 2-H, 3-H, 5-H, 6-H, 22-H, 23-H, 24-H, 25-H, 26-H), 6.73–6.66 (m, 1H, 4-H), 5.00 (s, 1H, 15-H), 2.59 (s, 3H, 10-H), 2.39 (s, 3H, 19-H), 1.65 (s, 3H, 17-H) ppm; ^13^C NMR (126 MHz, CDCl_3_): δ = 198.0 (C-18), 155.9 (C-12), 155.2 (C-11), 145.5 (C-7), 143.3 (C-13), 141.6 (C-9), 141.3 (C-21), 137.8 (C-1), 129.7 (C-3, C-5), 128.7 (C-23, C-25), 128.5 (C-2, C-6), 127.5 (C-24), 126.6 (C-22, C-26), 125.3 (C-20), 122.7 (C-4), 122.5 (C-14), 100.0 (C-8), 71.3 (C-16), 40.4 (C-15), 30.5 (C-19), 19.2 (C-17), 13.8 (C-10) ppm; MS (ESI, MeOH): *m*/*z*435 (100%, [M+H]^+^), 457 (22%, [M+Na]^+^); analysis calcd.for C_26_H_22_N_6_O (434.49): C 71.87, H 5.10, N 19.34; found: C 71.66, H 5.29, N 19.07.

##### 8-Acetyl-4-Amino-7-(4-methoxyphenyl)-3,9-dimethyl-1-phenyl-1,7-dihydropyrazolo[4,3-*e*] Pyrido[1,2-*a*]pyrimidine-6-carbonitrile (**7d**)

Yield: 74%; m.p. 160 °C; R_F_ = 0.33 (SiO_2_, CHCl_3_/MeOH, 95:5); UV–Vis (CHCl_3_): λ_max_ (log ε) = 273 nm (4.42); IR (ATR): ν = 2184m, 1607s, 1541s, 1509m, 1478m, 1355w, 1249m, 1174m, 1033m, 825w, 764m, 693m, 621w, 510s cm^−1^; ^1^H NMR (500 MHz, CDCl_3_): δ = 7.34–7.17 (m, 6H, 2-H, 3-H, 5-H, 6-H, 22-H, 26-H), 6.92–6.87 (m, 2H, 23-H, 25-H), 6.73 (m, 1H, 4-H), 4.92 (d, J = 1.0 Hz, 1H, 15-H), 3.77 (s, 3H, 27-H), 2.59 (s, 3H, 10-H), 2.38 (s, 3H, 19-H), 1.65 (s, 3H, 17-H) ppm; ^13^C NMR (126 MHz, CDCl_3_): δ = 198.1 (C-18), 159.0 (C-24), 155.7 (C-12), 155.1 (C-11), 145.5 (C-7), 143.2 (C-13), 141.3 (C-9), 137.9 (C-21), 133.5 (C-1), 129.8 (C-3, C-5), 128.6 (C-2, C-6), 127.7 (C-22, C-26), 125.5 (C-20), 122.8 (C-4), 122.5 (C-14), 114.1 (C-23, C-25), 100.0 (C-8), 71.8 (C-16), 55.5 (C-27), 39.8 (C-15), 30.5 (C-19), 19.2 (C-17), 13.8 (C-10) ppm; MS (ESI, MeOH): *m*/*z* 465 (100%, [M+H]^+^), 487 (40%, [M+Na]^+^); analysis calcd.for C_27_H_24_N_6_O_2_ (464.52): C 69.81, H 5.21, N 18.09; found: C 69.57, H 5.41, N 17.84.

##### 8-Acetyl-4-amino-7-(4-bromophenyl)-3,9-dimethyl-1-phenyl-1,7-dihydropyrazolo[4,3-*e*] Pyrido[1,2-*a*]pyrimidine-6-carbonitrile (**7e**)

Yield: 75%; m.p. 192 °C; R_F_ = 0.40 (SiO_2_, CHCl_3_/MeOH, 95:5); UV–Vis (CHCl_3_): λ_max_ (log ε) = 272 nm (4.27); IR (ATR): ν = 2186m, 1607s, 1541s, 1485m, 1396w, 1357w, 1223m, 1009m, 879w, 764m, 694m, 622w, 510m cm^−1^; ^1^H NMR (500 MHz, CDCl_3_): δ = 7.49 (d, J = 8.5 Hz, 2H, 23-H, 25-H), 7.32 (d, J = 7.4 Hz, 2H, 2-H, 6-H), 7.22 (d, J = 7.8 Hz, 2H, 3-H, 5-H), 7.16 (d, J = 8.5 Hz, 2H, 22-H, 26-H), 6.70 (d, J = 7.7 Hz, 1H, 4-H), 4.94 (s, 1H, 15-H), 2.58 (s, 3H, 10-H), 2.36 (s, 3H, 19-H), 1.67 (s, 3H, 17-H) ppm; ^13^C NMR (126 MHz, CDCl_3_): δ = 197.7 (C-18), 156.1 (C-12), 155.3 (C-11), 145.5 (C-7), 143.7 (C-13), 141.2 (C-21), 140.9 (C-9), 137.8 (C-1), 131.7 (C-23, C-25), 129.8 (C-3, C-5), 128.8 (C-2, C-6), 128.5 (C-22, C-26), 125.0 (C-20), 122.7 (C-14), 122.4 (C-4), 121.3 (C-24), 100.1 (C-8), 70.9 (C-16), 39.9 (C-15), 30.7 (C-19), 19.3 (C-17), 13.8 (C-10) ppm; MS (ESI, MeOH): *m*/*z* 513 (100%, [M+H]^+^); analysis calcd.for C_26_H_21_BrN_6_O (512.39): C 60.83, H 4.12, N 16.37; found: C 60.59, H 4.30, N 16.19.

##### 8-Acetyl-4-amino-7-(4-fluorophenyl)-3,9-dimethyl-1-phenyl-1,7-sihydropyrazolo[4,3-*e*] Pyrido[1,2-*a*]pyrimidine-6-carbonitrile (**7f**)

Yield: 80%; m.p. 259 °C; R_F_ = 0.38 (SiO_2_, CHCl_3_/MeOH, 95:5); UV–Vis (CHCl_3_): λ_max_ (log ε) = 273 nm (4.31), 289 nm (4.30); IR (ATR): ν = 2185m, 1610s, 1542s, 1478m, 1357w, 1223s, 1158m, 1013w, 862w, 764m, 695m, 623w, 513m cm^−1^; ^1^H NMR (500 MHz, CDCl_3_): δ = 7.32 (t, J = 7.5 Hz, 2H, 2-H, 6-H), 7.29–7.18 (m, 4H, 22-H, 23-H, 25-H, 26-H), 7.06 (t, J = 8.6 Hz, 2H, 3-H, 5-H), 6.72 (d, J = 7.2 Hz, 1H, 4-H), 4.96 (s, 1H, 15-H), 2.59 (s, 3H, 10-H), 2.37 (s, 3H, 19-H), 1.66 (s, 3H, 17-H) ppm; ^13^C NMR (126 MHz, CDCl_3_): δ = 197.6 (C-18), 161.9 (d, J = 246.6 Hz C-24), 155.8 (C-12), 155.0 (C-11), 145.3 (C-7), 143.2 (C-13), 141.0 (C-9), 137.6 (C-1), 137.2 (d, J = 3.,1 Hz, C-21), 129.5 (C-3, C-5), 128.4 (C-2, C-6), 128.0 (d, J = 7.9 Hz, C-22, C-26), 128.0 (22, 26), 125.1 (C-20), 122.4 (C-14), 122.2 (C-4), 115.3 (d, J = 21.4 Hz, C-23, C-25), 99.8 (C-8), 71.0 (C-16), 39.5 (C-15), 30.4 (C-19), 19.0 (C-17), 13.5 (C-10) ppm; MS (ESI, MeOH): *m*/*z* 453 (100%, [M+H]^+^), 475 (22%, [M+Na]^+^); analysis calcd.for C_26_H_21_FN_6_O (452.48): C 69.01, H 4.68,N 18.57; found: C 68.84, H 4.83, N 18.25.

##### 8-Acetyl-4-amino-3,9-dimethyl-7-(4-nitrophenyl)-1-phenyl-1,7-dihydropyrazolo[4,3-*e*] Pyrido[1,2-*a*]pyrimidine-6-carbonitrile (**7g**)

Yield: 58%; m.p. 164 °C; R_F_ = 0.31 (SiO_2_, CHCl_3_/MeOH, 95:5); UV–Vis (CHCl_3_): λ_max_ (log ε) = 270 nm (4.20); IR (ATR): ν = 2187m, 1608s, 1570m, 1542s, 1519m, 1477m, 1450w, 1344s, 1224m, 1191w, 1013w, 873w, 856w, 763m, 727w, 695m, 622w, 510mcm^−1^; ^1^H NMR (500 MHz, CDCl_3_): δ = 8.23 (d, J = 8.8 Hz, 2H, 23-H, 25-H), 7.48 (d, J = 8.2 Hz, 2H, 22-H, 26-H), 7.34 (t, J = 7.5 Hz, 2H, 2-H, 6-H), 7.20 (t, J = 7.8 Hz, 2H, 3-H, 5-H), 6.68 (d, J = 7.5 Hz, 1H, 4-H), 5.11 (s, 1H. 15-H), 2.58 (s, 3H, 10-H), 2.37 (s, 3H, 19-H), 1.70 (s, 3H, 17-H) ppm; ^13^C NMR (126 MHz, CDCl_3_): δ = 197.2 (C-18), 156.5 (C-12), 155.4 (C-11), 149.6 (C-21), 147.3 (C-24), 145.6 (C-7), 144.3 (C-13), 141.1 (C-9), 137.8 (C-1), 129.7 (C-3, C-5), 128.9 (C-2, C-6), 127.8 (C-22, C-26), 124.7 (C-20), 123.9 (C-23, C-25), 122.6 (C-14), 122.1 (C-4), 100.2 (C-8), 70.4 (C-16), 40.3 (C-15), 30.9 (C-19), 19.4 (C-17), 13.8 (C-10) ppm; MS (ESI, MeOH): *m*/*z* 480 (100%, [M+H]^+^), 502 (28%, [M+Na]^+^); analysis calcd.for C_26_H_21_N_7_O_3_ (479.49): C 65.13, H 4.41, N 20.45; found: C 64.97, H 4.68, N 20.22.

##### 8-Acetyl-4-amino-7-(3,4-dimethoxyphenyl)-3,9-dimethyl-1-phenyl-1,7-dihydropyrazolo[4,3-*e*] Pyrido[1,2-*a*]pyrimidine-6-carbonitrile (**7h**)

Yield: 72%; m.p. 260 °C; R_F_ = 0.33 (SiO_2_, CHCl_3_/MeOH, 95:5); UV–Vis (CHCl_3_): λ_max_ (log ε) = 286 nm (4.23); IR (ATR): ν = 2185m, 1607s, 1542s, 1514s, 1478m, 1253s, 1236s, 1137m, 1025m, 874w, 813w, 764m, 695m, 628w, 511m cm^−1^; ^1^H NMR (500 MHz, CDCl_3_): δ = 7.32–7.26 (m, 2H, 2-H, 6-H), 7.20 (t, J = 7.8 Hz, 2H, 3-H, 5-H), 6.91–6.61 (m, 4H, 4-H, 22-H, 23-H, 26-H), 4.92 (s, 1H, 15-H), 3.87 (s, 3H, 28-H), 3.83 (s, 3H, 27-H), 2.58 (s, 3H, 10-H), 2.38 (s, 3H, 19-H), 1.66 (s, 3H, 17-H) ppm; ^13^C NMR (126 MHz, CDCl_3_): δ = 198.4 (C-18), 156.3 (C-12), 155.4 (C-11), 149.7 (C-25), 148.9 (C-24), 145.6 (C-7), 143.4 (C-13), 141.6 (C-9), 138.2 (C-1), 134.5 (C-21), 129.9 (C-3, C-5), 128.9 (C-2, C-6), 125.8 (C-20), 123.0 (C-14), 122.8 (C-4), 118.4 (C-22), 111.1 (C-23), 110.9 (C-26), 100.3 (C-8), 71.9 (C-16), 56.5 (C-27, C-28), 40.3 (C-15), 30.8 (C-19), 19.4 (C-17), 14.0 (C-10) ppm; MS (ESI, MeOH): *m*/*z* 495 (100%, [M+H]^+^), 517 (82%, [M+Na]^+^); analysis calcd.for C_28_H_26_N_6_O_3_ (494.55): C 68.00, H 5.30, N 16.99; found: C 67.81, H 5.48, N 16.72.

##### 8-Acetyl-4-amino-7-(3,4-dichlorophenyl)-3,9-dimethyl-1-phenyl-1,7-dihydropyrazolo[4,3-*e*] Pyrido[1,2-*a*]pyrimidine-6-carbonitrile (**7i**)

Yield: 70%; m.p. 235 °C; R_F_ = 0.36 (SiO_2_, CHCl_3_/MeOH, 95:5); UV–Vis (CHCl_3_): λ_max_ (log ε) = 262 nm (4.29), 289 nm (4.25); IR (ATR): ν = 2183m, 1685m, 1600s, 1543s, 1474m, 1382w, 1353m, 1221m, 1183w, 1043w, 956w, 864m, 822w, 770m, 695m, 627m, 512s cm^−1^; ^1^H NMR (500 MHz, CDCl_3_): δ = 7.52 (d, J = 2.0 Hz, 1H, 25-H), 7.31–7.28 (m, 2H, 3-H, 5-H), 7.21–7.12 (m, 4H, 2-H, 6-H, 22-H, 23-H), 6.64 (d, J = 7.5 Hz, 1H, 4-H), 5.03 (s, 1H, 15-H), 2.61 (s, 3H, 10-H), 2.42 (s, 3H, 19-H), 1.50 (s, 3H, 17-H) ppm; ^13^C NMR (126 MHz, CDCl_3_): δ = 198.7 (C-18), 157.2 (C-12), 155.3 (C-11), 145.6 (C-7), 141.4 (C-9), 140.5 (C-13), 137.7 (C-1), 136.0 (C-21), 134.7 (C-26), 134.3 (C-24), 130.6 (C-25), 129.7 (C-2, C-6), 129.3 (C-22), 128.9 (C-3, C-5), 127.1 (C-23), 125.0 (C-20), 122.4 (C-4), 121.6 (C-14), 99.8 (C-8), 68.8 (C-16), 40.3 (C-15), 29.9 (C-19), 18.4 (C-17), 13.9 (C-10) ppm; MS (ESI, MeOH):*m*/*z* 503 (100%, [M+H]^+^), 525 (42%, [M+Na]^+^); analysis calcd.for C_26_H_20_Cl_2_N_6_O (502.38): C 62.04, H 4.00, N 16.70; found: C 61.87, H 45.29, N 16.52.

##### 8-Acetyl-4-amino-3,9-dimethyl-7-(5-nitro-2-thienyl)-1-phenyl-1,7-dihydropyrazolo[4,3-*e*] Pyrido[1,2-*a*]pyrimidine-6-carbonitrile (**7j**)

Yield: 74%; m.p. 262 °C; R_F_ = 0.60 (SiO_2_, CHCl_3_/MeOH, 95:5); UV–Vis (CHCl_3_): λ_max_ (logε) = 328 nm (3.86); IR (ATR): ν = 3434m, 2237m, 2195s, 1688m, 1650s, 1578w, 1549m, 1494s, 1420s, 1333s, 1262m, 1197m, 1112w, 955w, 816m, 759m, 688m, 660m, 635m, 562m, 510m cm^−1^; ^1^H NMR (500 MHz, CDCl_3_): δ = 7.69 (d, J = 4.2 Hz, 1H, 23-H), 7.51–7.46 (m, 2H, 2-H, 6-H), 7.44–7.38 (m, 2H, 3-H, 5-H), 7.28 (d, J = 7.2 Hz, 1H, 24-H), 6.67 (d, J = 4.2 Hz, 1H, 4-H), 4.86 (s, 1H, 15-H), 2.53 (s, 3H, 10-H), 2.23 (s, 3H, 19-H), 2.03 (s, 3H, 17-H) ppm; ^13^C NMR (126 MHz, CDCl_3_): δ = 198.0 (C-18), 157.6 (C-12), 157.5 (C-11), 153.2 (C-7), 149.3 (C-21), 142.5 (C-13), 138.0 (C-9), 136.3 (C-1), 131.0 (C-2, C-6), 130.6 (C-3, C-5), 129.5 (C-23), 124.8 (C-24), 123.7 (C-4), 118.9 (C-22), 115.9 (C-14), 95.3 (C-8), 65.1 (16), 36.5 (15), 30.3 (19), 17.7 (17), 13.8 (10) ppm; MS (ESI, MeOH): *m*/*z* 507 (100%, [M+Na]^+^); analysis calcd. for C_24_H_19_N_7_O_3_S (485.52): C 59.37, H 3.94, N 20.19, S 6.60; found: C 59.17, H 4.18, N 20.02, S 6.43.

##### 8-Acetyl-4-amino-3,9-dimethyl-7-(2-naphthyl)-1-phenyl-1,7-dihydropyrazolo[4,3-*e*] Pyrido[1,2-*a*]pyrimidine-6-carbonitrile (**7k**)

Yield: 65%; m.p. 270 °C; R_F_ = 0.49 (SiO_2_, CHCl_3_/MeOH, 95:5); UV–Vis (CHCl_3_): λ_max_ (log ε) = 270 nm (4.37); IR (ATR): ν = 3468w, 2181m, 1676m, 1654m, 1611m, 1543s, 1479m, 1357m, 1244m, 1222w, 951w, 879w, 762m, 754m, 695m, 621w, 515m, 479m cm^−1^; ^1^H NMR (500 MHz, CDCl_3_): δ = 7.89–7.80 (m, 3H, 23-H, 25-H, 28-H), 7.69 (t, J = 1.5 Hz, 1H, 30-H), 7.51–7.41 (m, 3H, 22-H, 26-H, 27-H), 7.22 (tt, J = 7.3, 1.0 Hz, 2H, 2-H, 6-H), 7.04 (t, J = 8.1 Hz, 2H, 3-H, 5-H), 6.60 (d, J = 7.5 Hz, 1H, 4-H), 5.23–5.06 (m, 1H, 15-H), 2.60 (s, 3H, 10-H), 2.45 (s, 3H, 19-H), 1.69 (s, 3H, 17-H) ppm; ^13^C NMR (126 MHz, CDCl_3_): δ = 198.1 (C-18), 155.7 (C-12), 155.2 (C-11), 145.6 (C-7), 143.7 (C-13), 141.3 (C-9), 139.0 (C-21), 137.8 (C-1), 133.3 (C-29), 132.8 (C-24), 129.7 (C-3, C-5), 128.6 (C-2, C-6), 128.6 (C-23), 128.0 (C-25), 127.8 (C-28), 126.7 (C-26), 126.3 (C-27), 125.3 (C-22), 125.1 (C-20), 124.8 (C-30), 122.6 (C-4), 122.4 (C-14), 99.9 (C-8), 71.5 (C-16), 40.7 (C-15), 30.6 (C-19), 19.4 (C-17), 13.8 (C-10) ppm; MS (ESI, MeOH): *m*/*z* 485 (100%, [M+H]^+^), 606 (24%, [M+Na]^+^); analysis calcd.for C_30_H_24_N_6_O (484.55): C 74.36, H 4.99, N 17.34; found: C 74.19, H 5.18, N 17.08.

##### 8-Acetyl-4-amino-3,9-dimethyl-1-phenyl-7-pyridin-3-yl-1,7-dihydropyrazolo[4,3-*e*] Pyrido[1,2-*a*]pyrimidine-6-carbonitrile (**7l**)

Yield: 68%; m.p. 306 °C; R_F_ = 0.44 (SiO_2_, CHCl_3_/MeOH, 95:5); UV–Vis (CHCl_3_): λ_max_(log ε) = 269 nm (4.34); IR (ATR): ν = 3398w, 2193m, 1651m, 1621s, 1570m, 1537s, 1474m, 1325m, 1232m, 1174m, 1025m, 957w, 877w, 759m, 716m, 689m, 623m, 572m, 511m cm^−1^; ^1^H NMR (500 MHz, CDCl_3_): δ = 8.58 (d, J = 4.0 Hz, 1H, 23-H), 8.52 (d, J = 2.4 Hz, 1H, 22-H), 7.79 (d, J = 7.9 Hz, 1H, 25-H), 7.42–7.27 (m, 5H, 2-H, 3-H, 5-H, 6-H, 24-H), 6.65 (d, J = 7.4 Hz, 1H, 4-H), 5.08 (s, 1H, 15-H), 2.58 (s, 3H, 10-H), 2.37 (s, 3H, 19-H), 1.70 (s, 3H, 17-H) ppm; ^13^C NMR (126 MHz, CDCl_3_): δ = 196.8 (C-18), 156.3 (C-12), 155.1 (C-11), 147.2 (C-23), 147.1 (C-22), 145.3 (C-7), 144.3 (C-13), 140.9 (C-9), 137.5 (C-1), 135.8 (C-25), 129.7 (C-3, C-5), 128.7 (C-2, C-6), 124.2 (C-20), 123.7 (C-21), 122.4 (C-4), 121.8 (C-14), 100.0 (C-8), 69.8 (C-16), 38.1 (C-15), 30.7 (C-19), 19.1 (C-17), 13.5 (C-10) ppm; MS (ESI, MeOH): *m*/*z* 436 (100%, [M+H]^+^); analysis calcd.for C_25_H_21_N_7_O (435.48): C 68.95, H 4.86, N 22.51; found: C 68.80, H 4.97, N 22.31.

### 3.3. Cytotoxicity Assay (SRB Assay)

The cell lines were obtained from Department of oncology (MartinLutherUniversity Halle-Wittenberg). Cultures were maintained as monolayers in RPMI 1640 medium with l-glutamine (Capricorn Scientific GmbH, Ebsdorfergrund, Germany) supplemented with 10% heat-inactivated fetal bovine serum (Sigma-Aldrich Chemie GmbH, Steinheim, Germany) and penicillin/streptomycin (1%, Capricorn Scientific GmbH, Ebsdorfergrund, Germany) at 37 °C in a humidified atmosphere with 5% CO_2_.

The cytotoxicity of the compounds was evaluated using the sulforhodamine-B (Kiton-Red S, ABCR: Karlsruhe, Germany) microculture colorimetric assay using confluent cells in 96-well plates with the seeding of the cells on day 0,by applying appropriate cell densities to prevent confluence of the cells during the period of the experiment. On day 1, the cells were treated with six different concentrations (1, 3, 7, 12, 20 and 30 μM); thereby, the final concentration of DMSO was always <0.5%, generally regarded as non-toxic to the cells. On day 4, the supernatant medium was discarded; the cells were fixed with 10% trichloroacetic acid. After another day at 4 °C, the cells were washed in a strip washer and dyed with the SRB solution (100 μL, 0.4% in 1% acetic acid) for about 20 min to be followed by washing of the plates (four times, 1% acetic acid) and air-drying overnight. Furthermore, Tris base solution (200 μL, 10 mM) was added to each well, and absorbance was measured at λ = 570 nm employing a reader (96 wells, Tecan Spectra, Crailsheim, Germany). The EC_50_ values were averaged from three independent experiments performed each in triplicate, calculated from semi-logarithmic dose–response curves applying a non-linear four-parameter Hill-slope equation (GraphPad Prism5 vom Graphpad Software Inc., San Diego, USA; the variables top and bottom were set to 100 and 0, respectively).

## 4. Conclusions

In summary, a new class of pyrazolo[4,3-*e*]pyrido[1,2-*a*]pyrimidine derivatives was designed, synthesized, characterized and evaluated for its cytotoxic activity toward five human cancer cell lines A375, HT-29, MCF-7, A2780, FaDu, as well as non-malignant NIH 3T3 and HEK293. Compound **7e** displayed a noteworthy cytotoxic effect toward all cancer cell lines. An SAR study demonstrated that fused heterocyclespyrazole, pyridine and pyrimidine and group linked to the aryl moiety E in the *para* position, especially the presence of a bromine substituent, seem to play a crucial role in the cytotoxic activities. In addition, the molecular docking indicated that this class of heterocyclic molecules exhibits important binding energy and interaction with interesting residues of anticancer targets such as, DHFR, VEGFR2, HER-2/neu, hCA-IX, CDK6 and LOX5.

## Figures and Tables

**Table 1:** Cytotoxicity of compounds **3**, **5**, **7a**–**l** against the human cancer cell lines A375, HT29, MCF-7, A2780, FaDu and non-malignant cells NIH 3T3 and HEK293 (n.d., not determined;n.s., not soluble under the conditions of the assay).

Compound	A375	HT29	MCF-7	A2780	FaDu	NIH 3T3	HEK293
**3**, **5**	>30	>30	>30	>30	>30	>30	>30
**7a**	19.3 ± 3.0	29.9 ± 1.4	17.2 ± 1.4	18.0 ± 3.0	>30	23.0 ± 1.4	15.8 ± 2.0
**7b**	12.9 ± 1.6	17.5 ± 1.8	12.2 ± 1.3	14.6 ± 2.2	22.3 ± 2.7	21.3 ± 0.9	12.4 ± 0.9
**7c**	19.3 ± 3.8	>30	19.5 ± 1.1	25.4 ± 2.5	>30	>30	7.7 ± 1.1
**7d**	21.5 ± 3.6	28.3 ± 3.5	18.4 ± 2.3	21.7 ± 3.9	>30	>30	16.7 ± 1.2
**7e**	9.4 ± 1.2	13.3 ± 1.8	9.2 ± 0.7	9.1 ± 1.6	13.5 ± 1.5	12.3 ± 0.7	6.6 ± 0.6
**7f**	18.1 ± 3.3	24.7 ± 4.4	19.2 ± 1.3	19.2 ± 3.9	>30	24.1 ± 2.8	14.7 ± 1.4
**7g**	16.2 ± 2.0	25.9 ± 3.4	15.7 ± 2.2	14.5 ± 2.2	>30	>30	17.0 ± 1.3
**7h**	22.1 ± 4.9	>30	25.1 ± 2.8	27.5 ± 8.1	>30	>30	24.0 ± 3.4
**7i**–**l**	n.s.	n.s.	n.s.	n.s.	n.s.	n.s.	n.s.
**DX**	n.d.	0.9 ± 0.01	1.1 ± 0.3	0.01 ± 0.01	n.d.	0.4 ± 0.07	n.d.

**Table 2:** Free binding energy (Kcal·mol^−1^^)^ of top-ranked active compound, **7e**, with the active site of different anticancer drug targets studied using AutoDock 4.2 software.

Compound	Free Binding Energy (Kcal·mol^−1^)					
	DHFR (5HQY)	VEGFR2 (5EW3)	HER-2 (3RCD)	hCA-IX (5FL4)	CDK6 (3NUP)	LOX5 (3V99)
**7e**	−8.5	−6.6	−7.3	−7.2	−7.1	−8.5
Ref. ligand	−8.3	−7.3	−7.2	−7.0	−6.8	−6.0

## Data Availability

The source data underlying tables and figures are available from the authors upon request.

## Supplementary Materials

Supplementary materials can be found at https://www.mdpi.com/article/10.3390/ijms221910258/s1.

## References

[1] Torre L.A., Bray F., Siegel R.L., Ferlay J., Lortet-Tieulent J., Jemal A. (2015). Global Cancer Statistics, 2012. CA-Cancer J. Clin..

[2] Isakoff S.J. (2010). Triple-Negative Breast Cancer Role of Specific Chemotherapy Agents. Cancer J..

[3] Nossier E.S., Fahmy H.H., Khalifa N.M., El-Eraky W.I., Baset M.A. (2017). Design and Synthesis of Novel Pyrazole-Substituted Different Nitrogenous Heterocyclic Ring Systems as Potential Anti-Inflammatory Agents. Molecules.

[4] Chekir S., Debbabi M., Regazzetti A., Dargere D., Laprevote O., Ben Jannet H., Gharbi R. (2018). Design, synthesis and biological evaluation of novel 1,2,3-triazole linked coumarinopyrazole conjugates as potent anticholinesterase, anti-5-lipoxygenase, anti-tyrosinase and anti-cancer agents. Bioorg. Chem..

[5] El-Naggar M., Hassan A.S., Awad H.M., Mady M.F. (2018). Design, Synthesis and Antitumor Evaluation of Novel Pyrazolopyrimidines and Pyrazoloquinazolines. Molecules.

[6] Somakala K., Tariq S., Amir M. (2019). Synthesis, evaluation and docking of novel pyrazolo pyrimidines as potent p38 alpha MAP kinase inhibitors with improved anti-inflammatory, ulcerogenic and TNF-alpha inhibitory properties. Bioorg. Chem..

[7] Rahmouni A., Souiei S., Belkacem M.A., Romdhane A., Bouajila J., Ben Jannet H. (2016). Synthesis and biological evaluation of novel pyrazolopyrimidines derivatives as anticancer and anti-5-lipoxygenase agents. Bioorg. Chem..

[8] Rahmouni A., Romdhane A., Ben Said A., Majouli K., Ben Jannet H. (2014). Synthesis of new pyrazole and antibacterial pyrazolopyrimidine derivatives. Turk. J. Chem..

[9] De Vita D., Pandolfi F., Cirilli R., Scipione L., Di Santo R., Friggeri L., Mori M., Fiorucci D., Maccari G., Christopher R.S.A. (2016). Discovery of in vitro antitubercular agents through in silico ligand-based approaches. Eur. J. Med. Chem..

[10] Al-Aldiwish W.M., Shtewi F.A., Ashrif M.M., Ibrahim D.M. (2017). Synthesis, biolovical activity and cytotoxicity of new fused pyrazolo[1,5-a]pyrimidine from 5-aminopyrazole incoroporated with p-chloroaniline. Am. J. Heterocycl. Chem..

[11] Alharthy R.D. (2020). Design and Synthesis of Novel Pyrazolo[3,4-d]Pyrimidines: In Vitro Cytotoxic Evaluation and Free Radical Scavenging Activity Studies. Pharm. Chem. J..

[12] Fouda A.M., Abbas H.A.S., Ahmed E.H., Shati A.A., Alfaifi M.Y., Elbehairi S.E.I. (2019). Synthesis, In Vitro Antimicrobial and Cytotoxic Activities of Some New Pyrazolo[1,5-a]pyrimidine Derivatives. Molecules.

[13] Husseiny E.M. (2020). Synthesis, cytotoxicity of some pyranzoles and pyrazolo[1,5-a]pyrimidines bearing benzothiazole moiety amd investigation of their mechanism of action. Bioorg. Chem..

[14] Zhang H.J., Wang S.B., Wen X., Li J.Z., Quan Z.S. (2016). 5Y Design, synthesis, and evaluation of the anticonvulsant and antidepressant activities of pyrido[2,3-d]pyrimidine derivatives. Med. Chem. Res..

[15] Abdelaziz O.A., El Husseiny W.M., Selim K.B., Eisa H.M. (2019). Dihydrofolate reductase inhibition effect of 5-substituted pyrido[2,3-d] pyrimidines: Synthesis, antitumor activity and molecular modeling study. Bioorg. Chem..

[16] Ibrahim D.A., Ismail N.S.M. (2011). Design, synthesis and biological study of novel pyrido[2,3-d]pyrimidine as anti-proliferative CDK2 inhibitors. Eur. J. Med. Chem..

[17] Hanafy F.I. (2011). Synthesis and antifungal activity of some new pyrido[2,3-d]pyrimidines. Eur. J. Chem..

[18] Gfesser G.A., Bayburt E.K., Cowart M., Di Domenico S., Gomtsyan A., Lee C.H., Stewart A.O., Jarvis M.F., Kowaluk E.A., Bhagwat S.S. (2003). Synthesis and structure-activity relationships of 5-heteroatom-substituted pyridopyrimidines as adenosine kinase inhibitors. Eur. J. Med. Chem..

[19] Moreno E., Plano D., Lamberto I., Font M., Encio I., Palop J.A., Sanmartin C. (2012). Sulfur and selenium derivatives of quinazoline and pyrido[2,3-d]pyrimidine: Synthesis and study of their potential cytotoxic activity in vitro. Eur. J. Med. Chem..

[20] Sanmartin C., Echeverria M., Mendivil B., Cordeu L., Cubedo E., Garcia-Foncillas J., Font M., Palop J.A. (2005). Synthesis and biological evaluation of new symmetrical derivatives as cytotoxic agents and apoptosis inducers. Bioorg. Med. Chem..

[21] Alam R., Alam A., Panda A.K. (2018). Design, synthesis and cytotoxicity evaluation of pyrazolyl pyrazoline and pyrazolyl aminopyrimidine derivatives as potential anticancer agents. Med. Chem. Res..

[22] Lee J., Kim K.H., Jeong S. (2011). Discovery of a novel class of 2-aminopyrimidines as CDK1 and CDK2 inhibitors. Bioorg. Med. Chem. Lett..

[23] Sabour R., Harras M.F., Mehany A.B.M. (2019). Design, Synthesis, Cytotoxicity Screening and Molecular Docking of New 3- Cyanopyridines as Survivin inhibitors and Apoptosis Inducers. Bioorg. Chem..

[24] Al-Refai M., Ibrahim M.M., Nurul Azmi M., Osman H., Abu Bakar M.H., Geyer A. (2019). The Synthesis, Characterization, Cytotoxic Activity Assessment and Structure-Activity Relationship of 4-Aryl-6-(2,5-dichlorothiophen-3-yl)-2-methoxypyridine-3-carbonitriles. Molecules.

[25] Ragab F.A., Nissan Y.M., Seif E.M., Maher A., Arafa R.K. (2020). Synthesis and in vitro investigation of novel cytotoxic pyrimidine and pyrazolopyrimidne derivatives showing apoptotic effect. Bioorg. Chem..

[26] Haiba M.E., Al-Abdullah E.S., Ahmed N.S., Ghabbour H.A., Awad H.M. (2019). Efficient and easy synthesis of new Benzo[h]chromene and Benzo[h] quinoline derivatives as a new class of cytotoxic agents. J. Mol. Struct..

[27] Debbabi M., Nimbarte V.D., Chekir S., Chortani S., Romdhane A., Ben Jannet H. (2019). Design and synthesis of novel potent anticoagulant and anti-tyrosinase pyranopyrimidines and pyranotriazolopyrimidines: Insights from molecular docking and SAR analysis. Bioorg. Chem..

[28] Horchani M., Della Sala G., Caso A., D’Aria F., Esposito G., Laurenzana I., Giancola C., Costantino V., Ben Jannet H., Romdhane A. (2021). Molecular Docking and Biophysical Studies for Antiproliferative Assessment of Synthetic Pyrazolo-Pyrimidinones Tethered with Hydrazide-Hydrazones. Int. J. Mol. Sci..

[29] Romdhane A., Martin M.T., Said A., Jabrane A., Jannet H. (2012). Synthesis of new naphto[2,1-b]pyrano[3,2-e][1,2,4]triazolo[1,5-c]pyrimidine derivatives and their evaluation as acetylcholinesterase inhibitors. J. Soc. Chim. Tun..

[30] Horchani M., Hajlaoui A., Harrath A.H., Mansour L., Ben Jannet H., Romdhane A. (2020). New pyrazolo-triazolo-pyrimidine derivatives as antibacterial agents: Design and synthesis, molecular docking and DFT studies. J. Mol. Struct..

[31] Aliwaini S., Abu Thaher B., Al-Masri I., Shurrab N., El-Kurdi S., Schollmeyer D., Qeshta B., Ghunaim M., Csuk R., Laufer S. (2021). Design, Synthesis and Biological Evaluation of Novel Pyrazolo[1,2,4]triazolopyrimidine Derivatives as Potential Anticancer Agents. Molecules.

[32] Saeed A., Hussain H., Shamraiz U., Rehman N.U., Khan H.Y., Badshah A., Heller L., Csuk R., Ali M., Khan A. (2018). Synthesis of new triterpenic monomers and dimers as potential antiproliferative agents and their molecular docking studies. Eur. J. Med. Chem..

[33] Irina A.T., Alexey V.N., Daria V.E., Victoria V.G. (2018). Synthesis, cytotoxic evaluation, and molecular docking studies of the semi-synthetic “triterpenoid-steroid” hybrids. Steroids.

[34] Ruddarraju R.R., Murugulla A.C., Kotla R., Tirumalasetty M.C.B., Wudayagiri R., Donthabakthuni S., Maroju R., Baburao K., Parasa L.S. (2016). Design, synthesis, anticancer, antimicrobial activities and molecular docking studies of theophylline containing acetylenes and theophylline containing 1,2,3-triazoles with variant nucleoside derivatives. Eur. J. Med. Chem..

[35] Berman H.M., Westbrook J., Feng Z., Gilliland G., Bhat T.N., Weissig H., Shindyalov I.N., Bourne P.E. (2000). The Protein Data Bank. Nucleic Acids Res..

[36] Bold G., Schnell C., Furet P., McSheehy P., Bruggen J., Mestan J., Manley P.W., Druckes P., Burglin M., Durler U. (2016). Littlewood-Evans, A., A Novel Potent Oral Series of VEGFR2 Inhibitors Abrogate Tumor Growth by Inhibiting Angiogenesis. J. Med. Chem..

[37] Ishikawa T., Seto M., Banno H., Kawakita Y., Oorui M., Taniguchi T., Ohta Y., Tamura T., Nakayama A., Miki H. (2011). Design and Synthesis of Novel Human Epidermal Growth Factor Receptor 2 (HER2)/Epidermal Growth Factor Receptor (EGFR) Dual Inhibitors Bearing a Pyrrolo[3,2-d]pyrimidine Scaffold. J. Med. Chem..

[38] Leitans J., Kazaks A., Balode A., Ivanova J., Zalubovskis R., Supuran C.T., Tars K. (2015). Efficient Expression and Crystallization System of Cancer-Associated Carbonic Anhydrase Isoform IX. J. Med. Chem..

[39] Cho Y.S., Borland M., Brain C., Chen C.H.T., Cheng H., Chopra R., Chung K., Groarke J., He G., Hou Y. (2010). 4-(Pyrazol-4-yl)-pyrimidines as Selective Inhibitors of Cyclin-Dependent Kinase 4/6. J. Med. Chem..

[40] Gilbert N.C., Rui Z., Neau D.B., Waight M.T., Bartlett S.G., Boeglin W.E., Brash A.R., Newcomer M.E. (2012). Conversion of human 5-lipoxygenase to a 15-lipoxygenase by a point mutation to mimic phosphorylation at Serine-663. FASEB J..

