# Supplementary material for: Synthesis and In Silico Docking of New Pyrazolo[4,3-e]pyrido[1,2-a]pyrimidine-based Cytotoxic Agents
Source: Int J Mol Sci. 2021 Sep 23;22(19):10258. doi: 10.3390/ijms221910258 (PMC8508785; doi:10.3390/ijms221910258)

Supplementary material

Synthesis and *in silico* docking of new pyrazolo [*4,3-e*] pyrido [*1,2-a*] pyrimidine-based cytotoxic agents

Mabrouk Horchani[1], Niels V. Heise[2], Sophie Hoenke[2], René Csuk[2,*], Abdel Halim Harrath[3], Hichem Ben Jannet[1,*], Anis Romdhane[1]

[1] Laboratory of Heterocyclic Chemistry, Natural Products and Reactivity, Medicinal Chemistry and Natural Products (LR11ES39), Faculty of sciences Monastir, University of Monastir, 5000 Monastir, Tunisia

[2] Organic Chemistry, Martin-Luther University Halle-Wittenberg, Kurt-Mothes Str. 2, D-06120 Halle (Saale), Germany

[3] King Saud University, Department of Zoology, College of Science, Riyadh, Saudi Arabia

1. *General*

NMR spectra were recorded using the Agilent spectrometers DD2 500 MHz and VNMRS 400 MHz (δgiven in ppm, J in Hz; typical experiments: APT, H-H-COSY, HMBC, HSQC, NOESY), MSspectra were taken on an Advion Expression CMS instrument. TLC was performed on silica gel (Macherey-Nagel, detection with cerium molybdatereagent); melting points are uncorrected (Leica hot stage microscope, or BUCHI melting point M-565), and elemental analyseswere performed on a Foss-Heraeus Vario EL (CHNS) unit. IR spectra were recorded on aPerkin Elmer FT-IR spectrometer Spectrum 1000or on a Perkin-Elmer Spectrum Two (UATR Two Unit). The solvents were driedaccording to usual procedures.

2. *NMR Spectra*

Spectra of 3:

^{1}H NMR (500 MHz, $CDCl_3$):

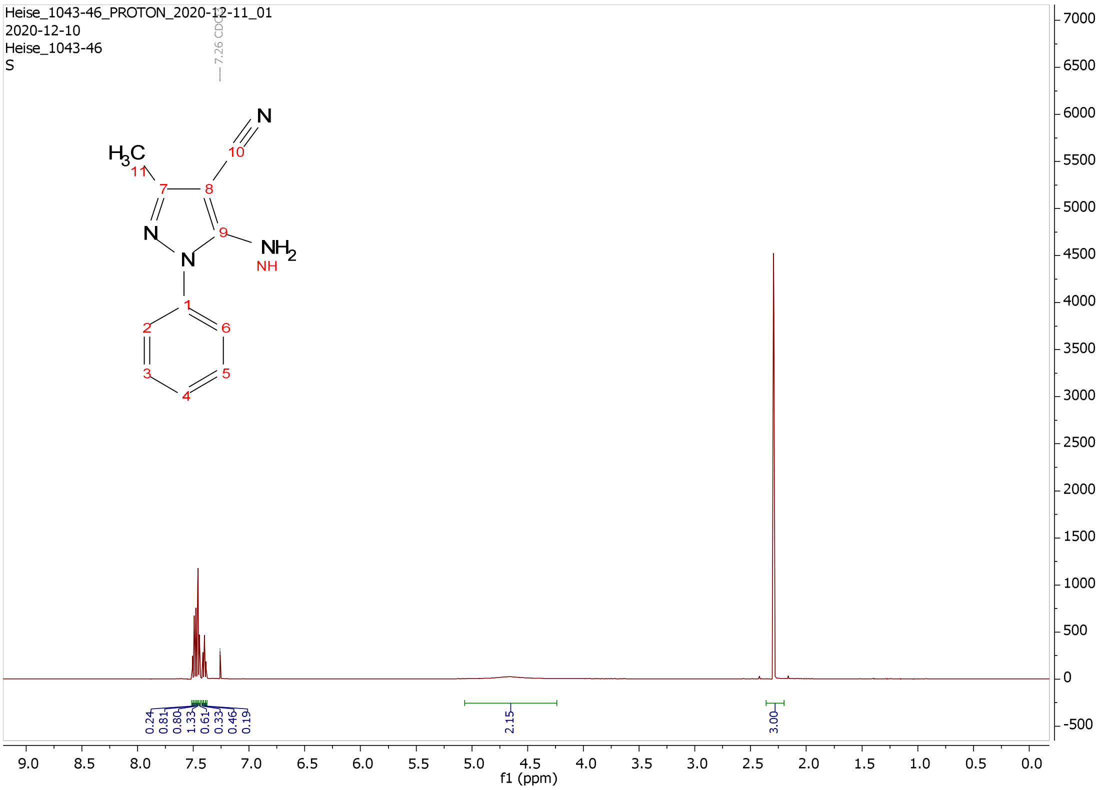

^{13}C NMR (126 MHz, $CDCl_3$):

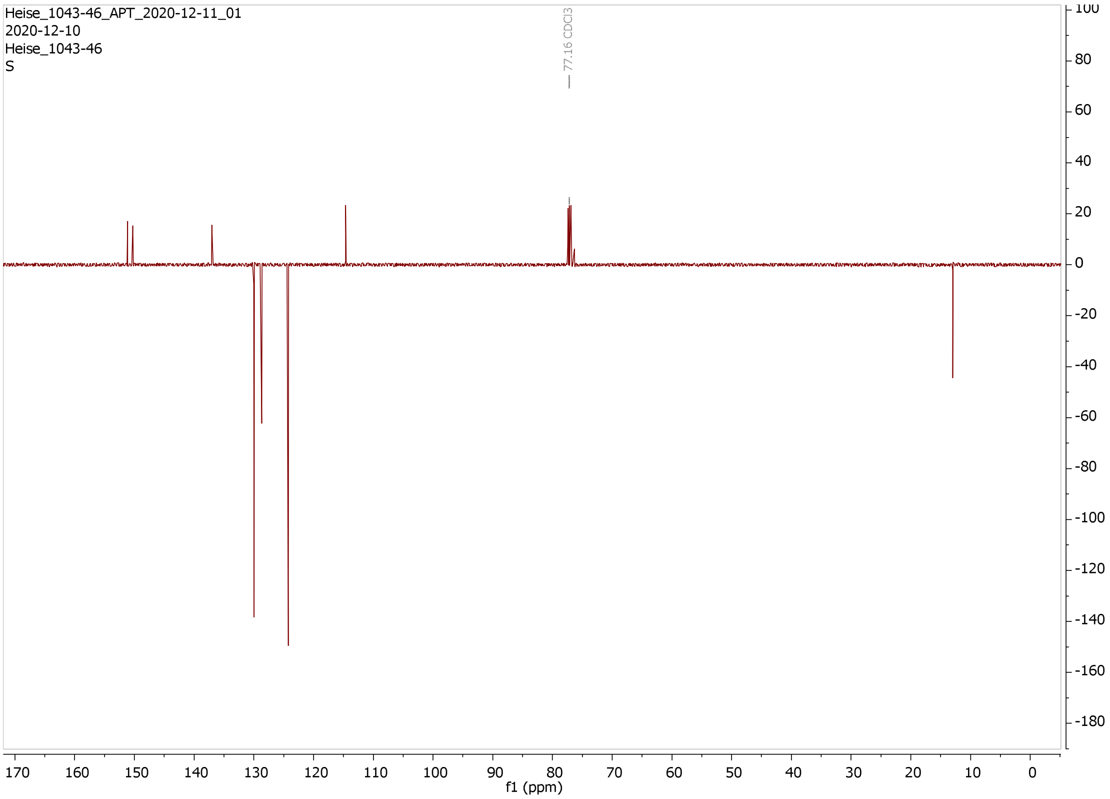

IR spectrum (ATR):

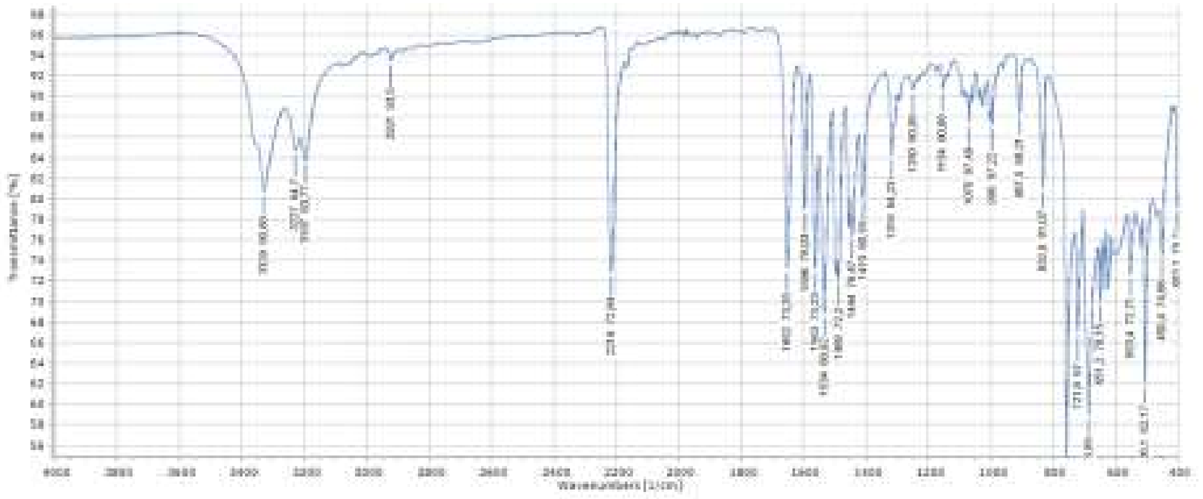

Spectra of 5:

^{1}H NMR (500 MHz, $CDCl_3$):

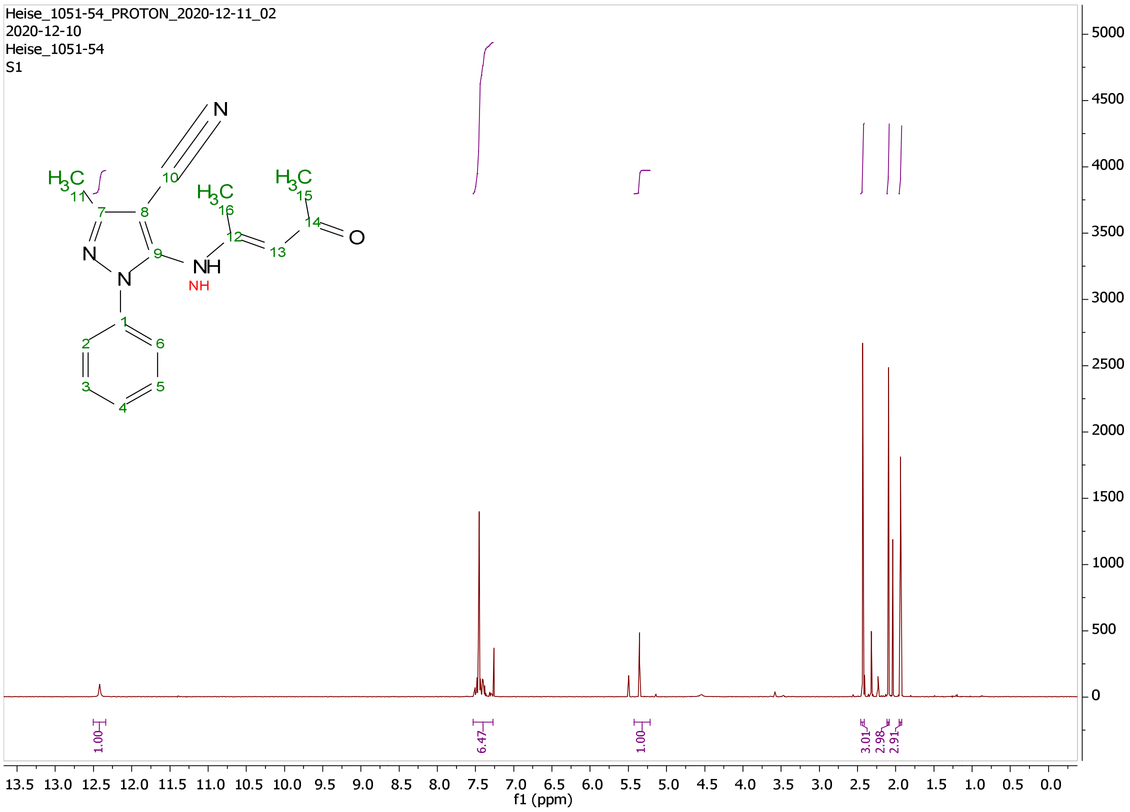

^{13}C NMR (126 MHz, $CDCl_3$):

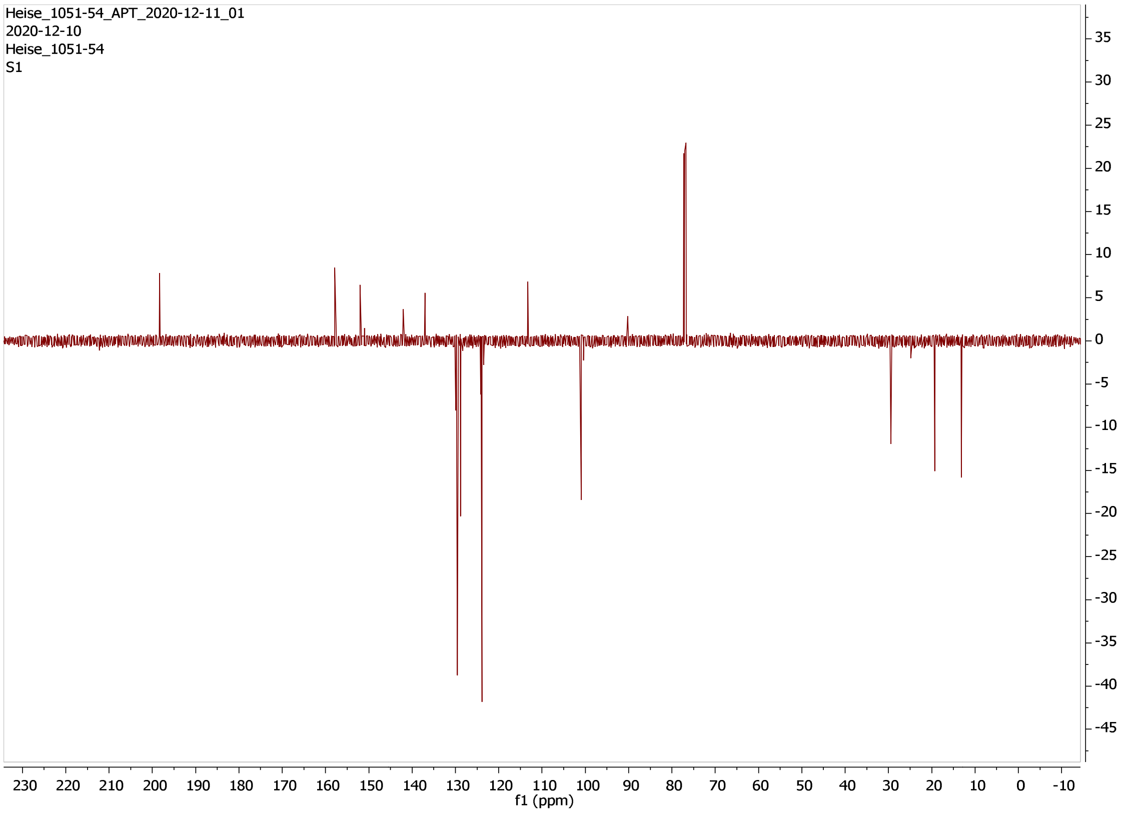

IR spectrum (ATR):

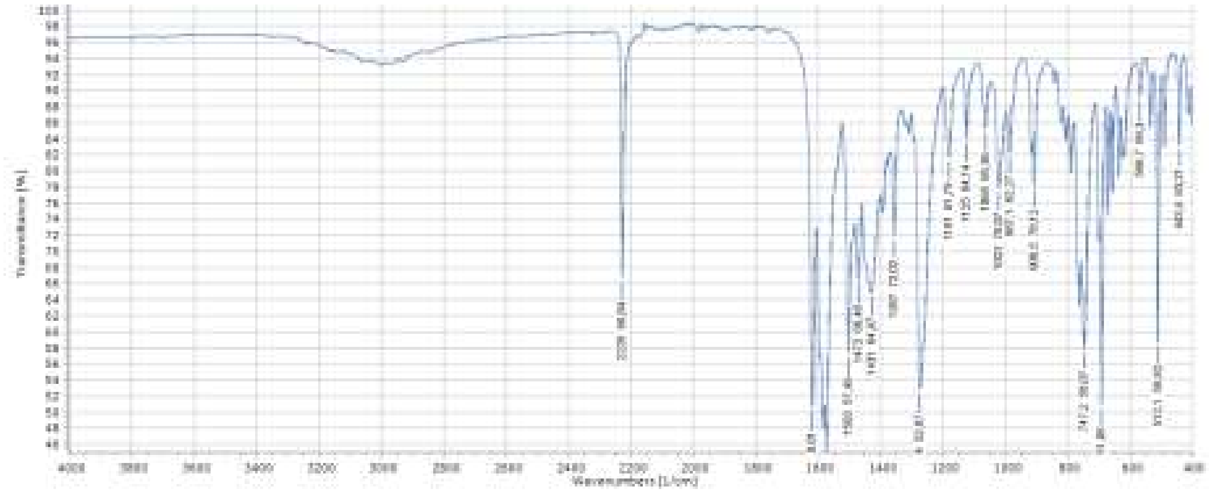

Spectra of 7a:

^{1}H NMR (500 MHz, $CDCl_3$):

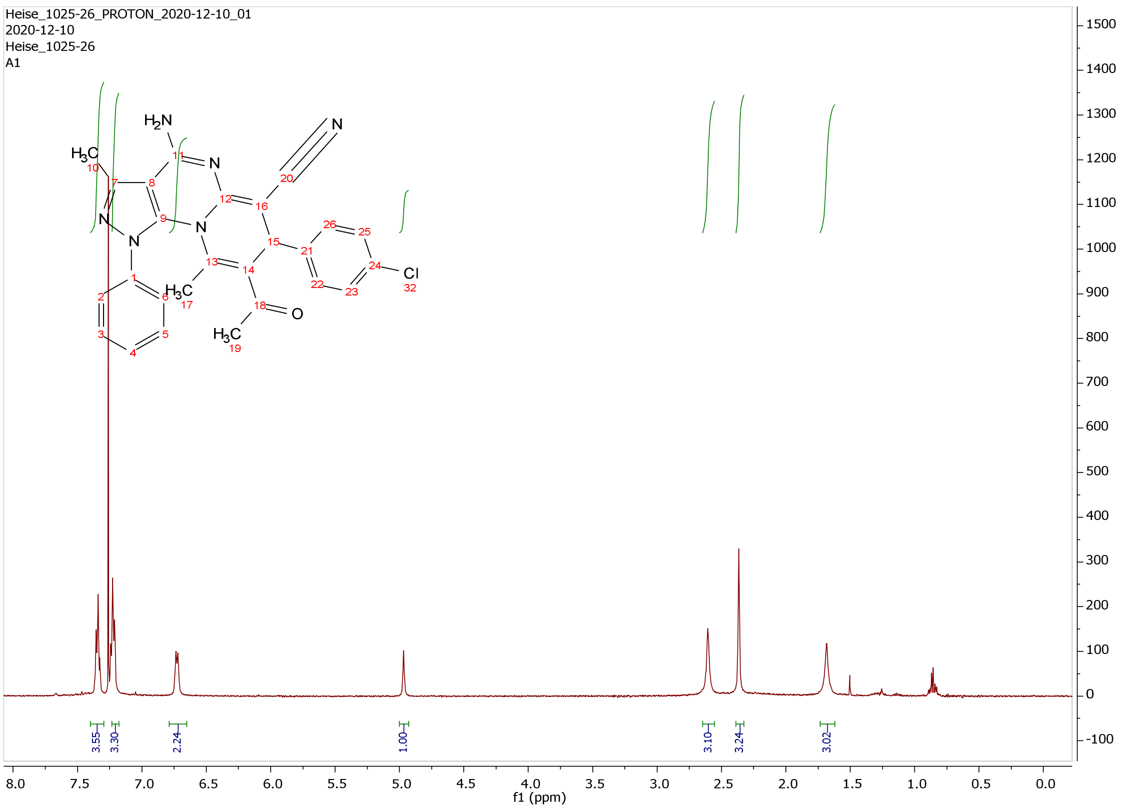

^{13}C NMR (126 MHz, $CDCl_3$):

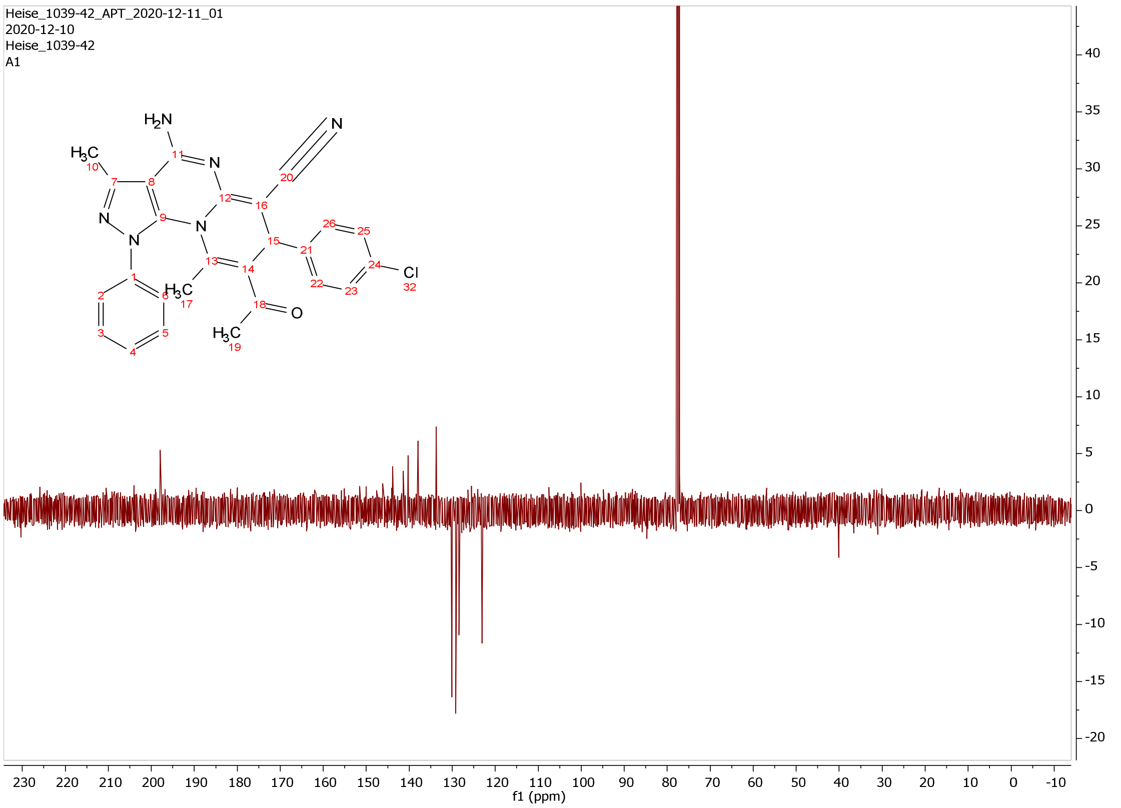

IR spectrum (ATR):

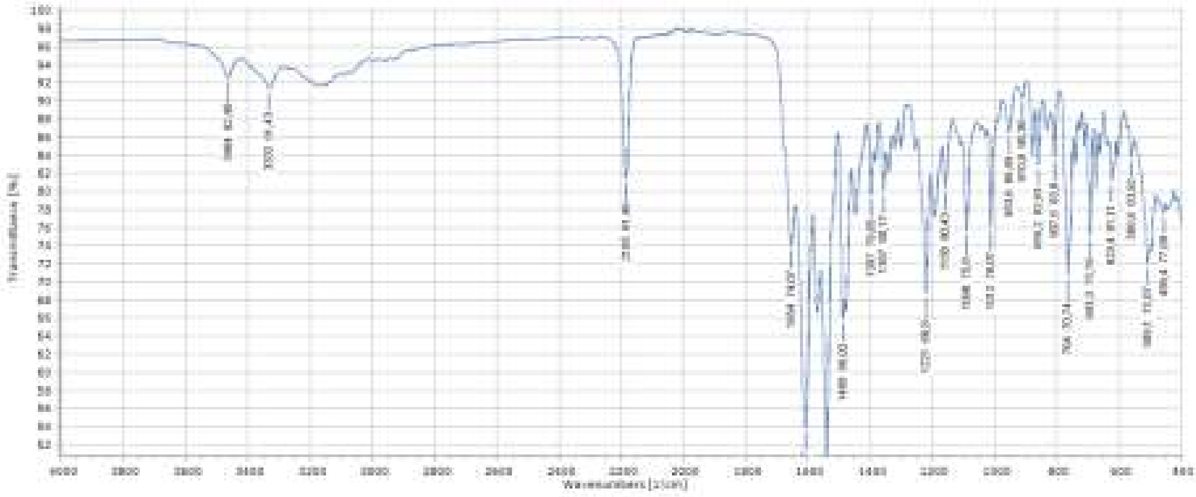

Spectra of 7b:

^{1}H NMR (500 MHz, $CDCl_3$):

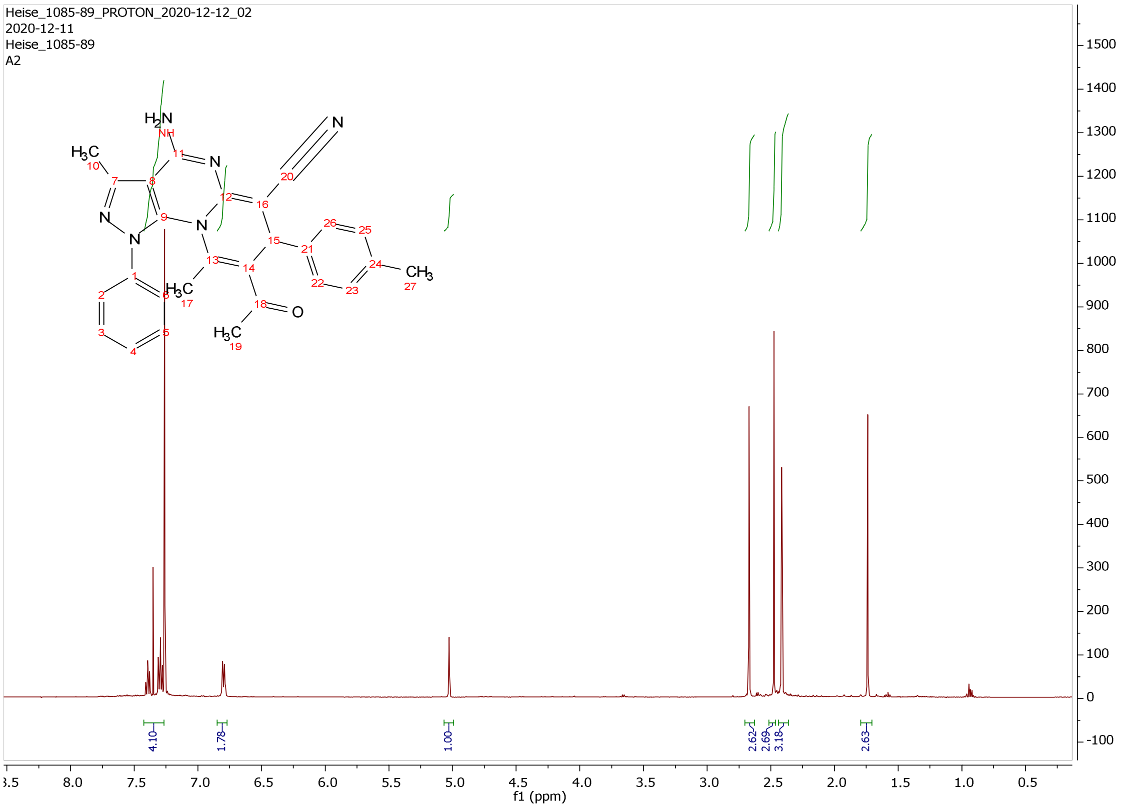

^{13}C NMR (126 MHz, $CDCl_3$):

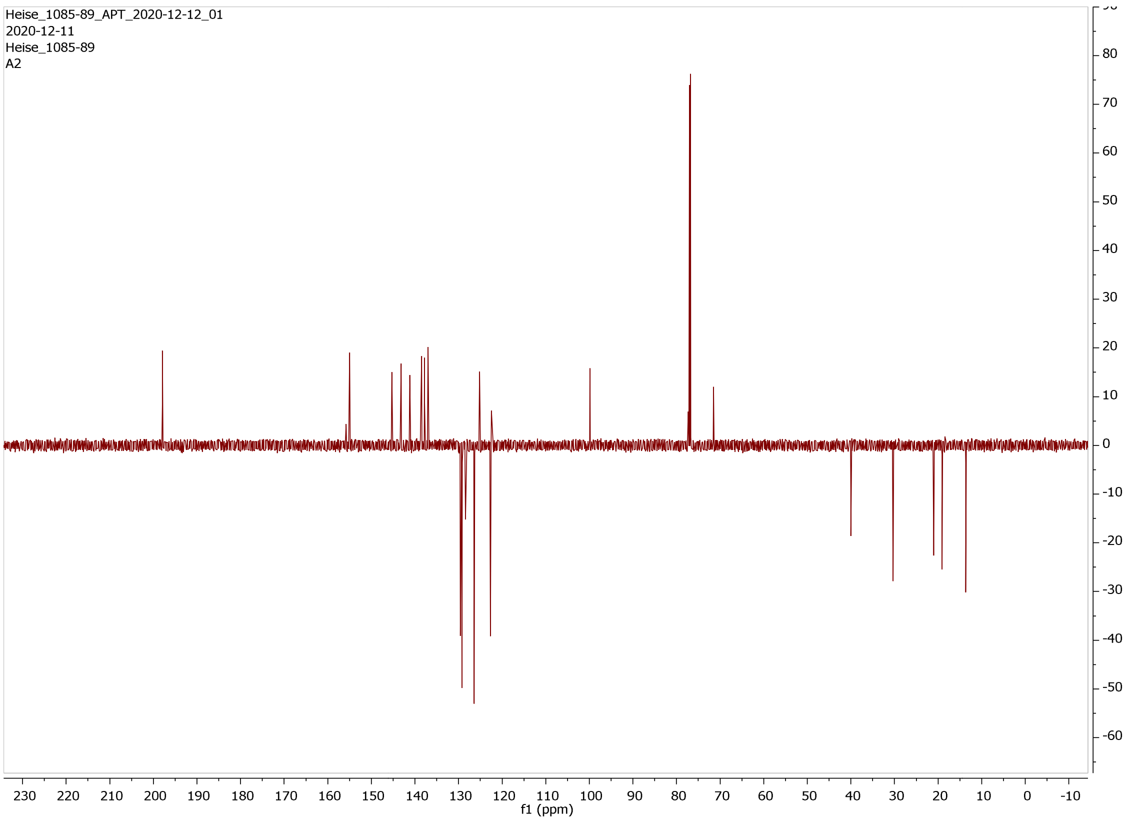

IR spectrum (ATR):

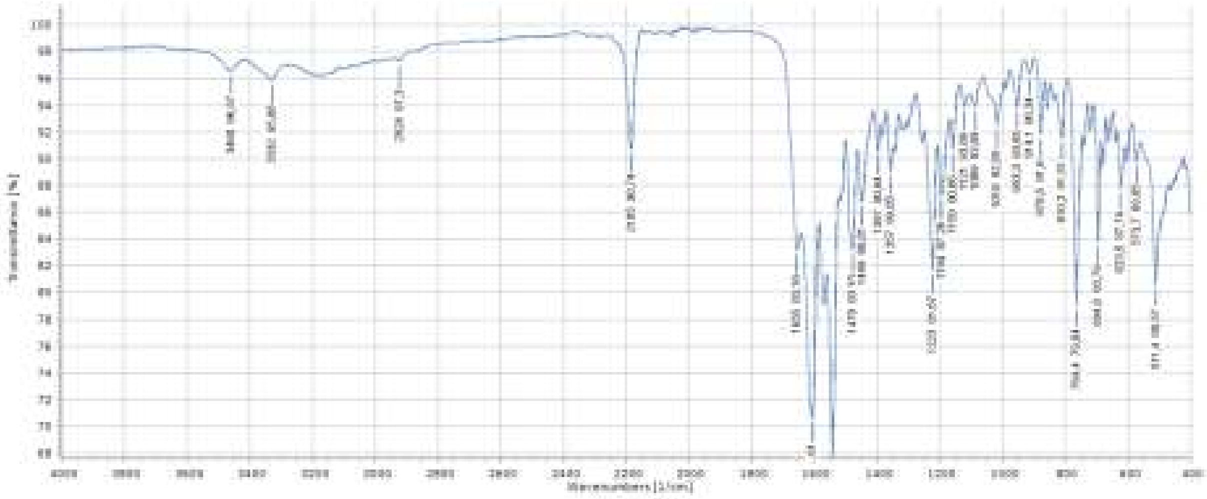

Spectra of 7c:

^{1}H NMR (500 MHz, $CDCl_3$):

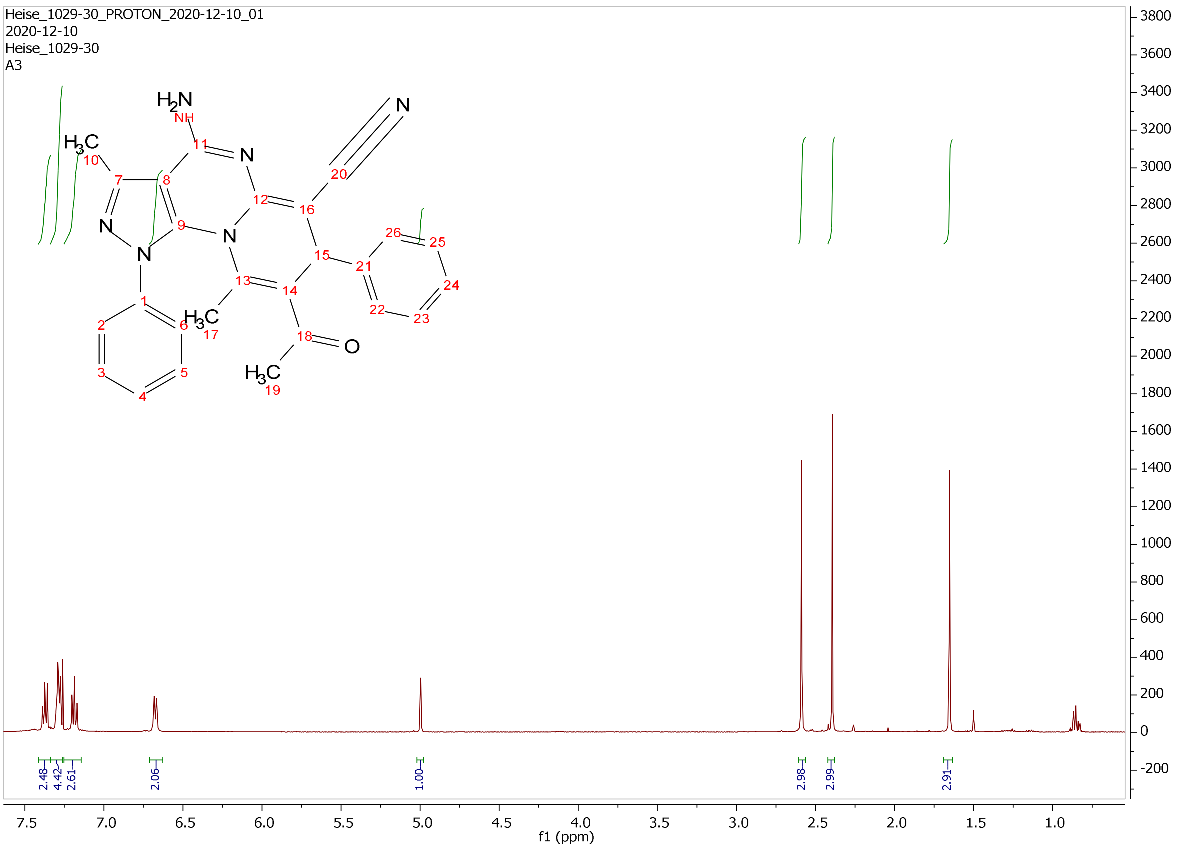

^{13}C NMR (126 MHz, $CDCl_3$):

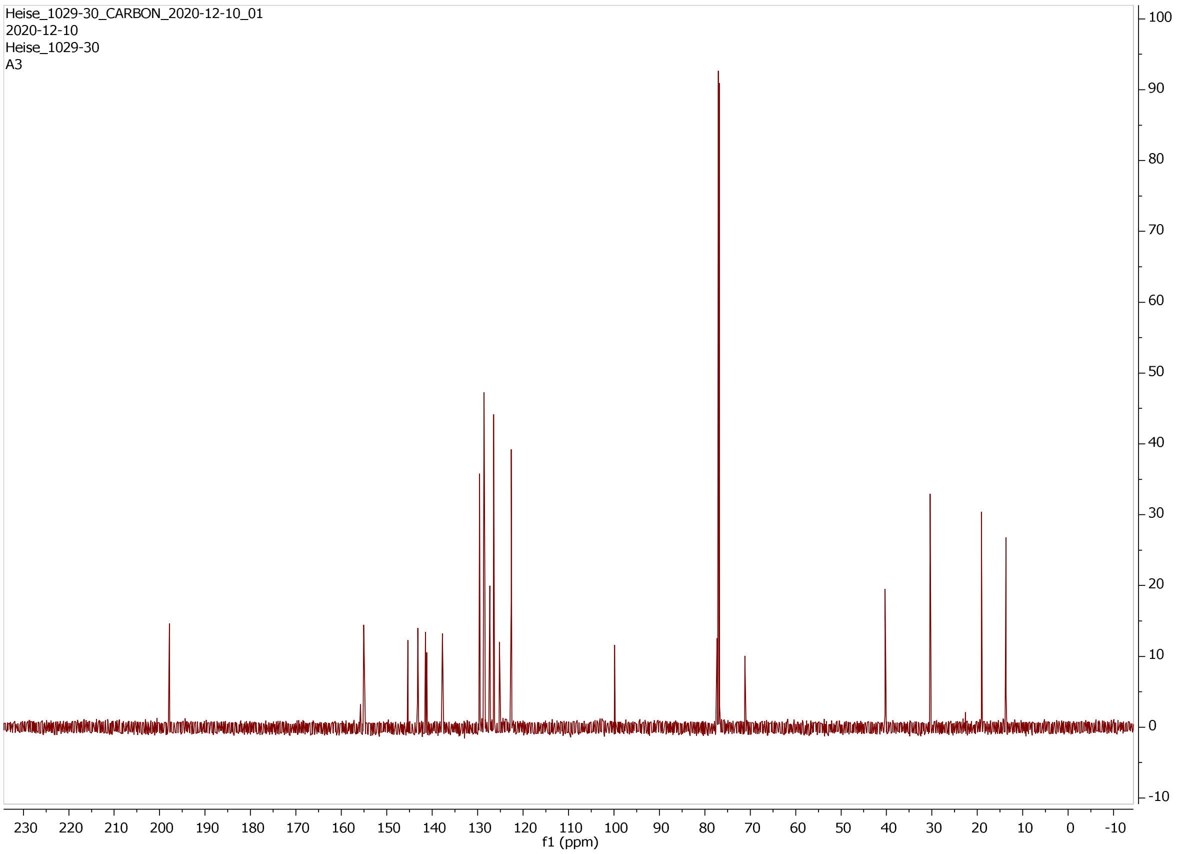

IR spectrum (ATR):

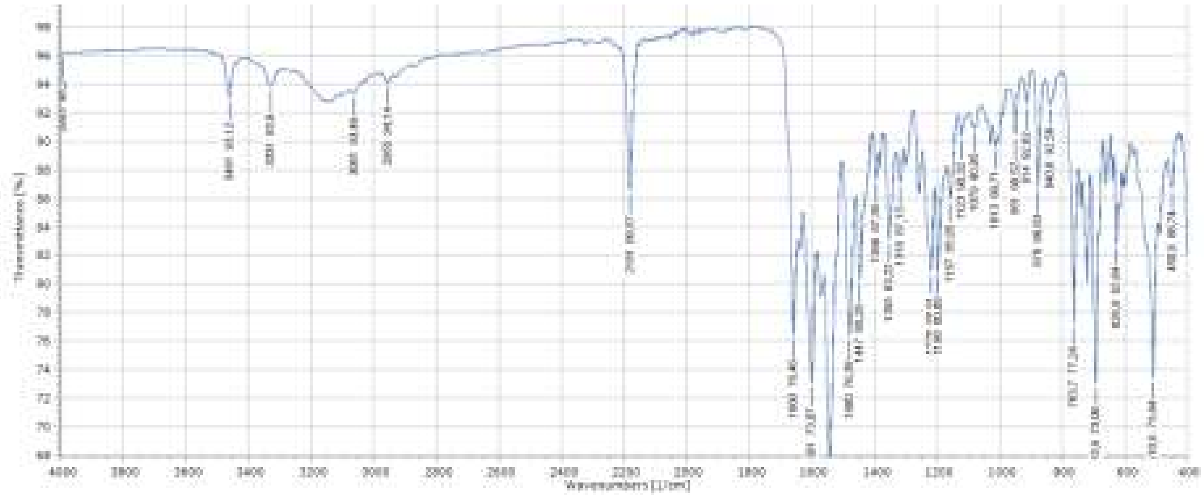

Spectra of 7d:

^{1}H NMR (500 MHz, $CDCl_3$):

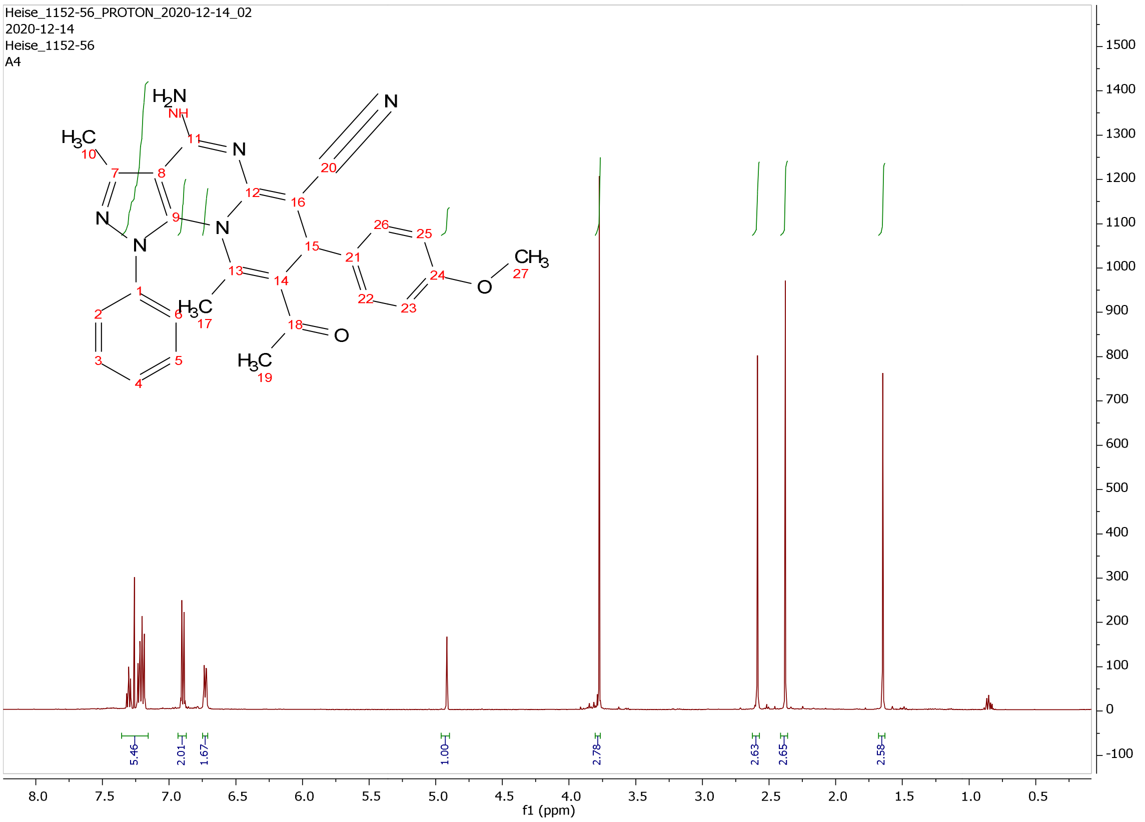

^{13}C NMR (126 MHz, $CDCl_3$):

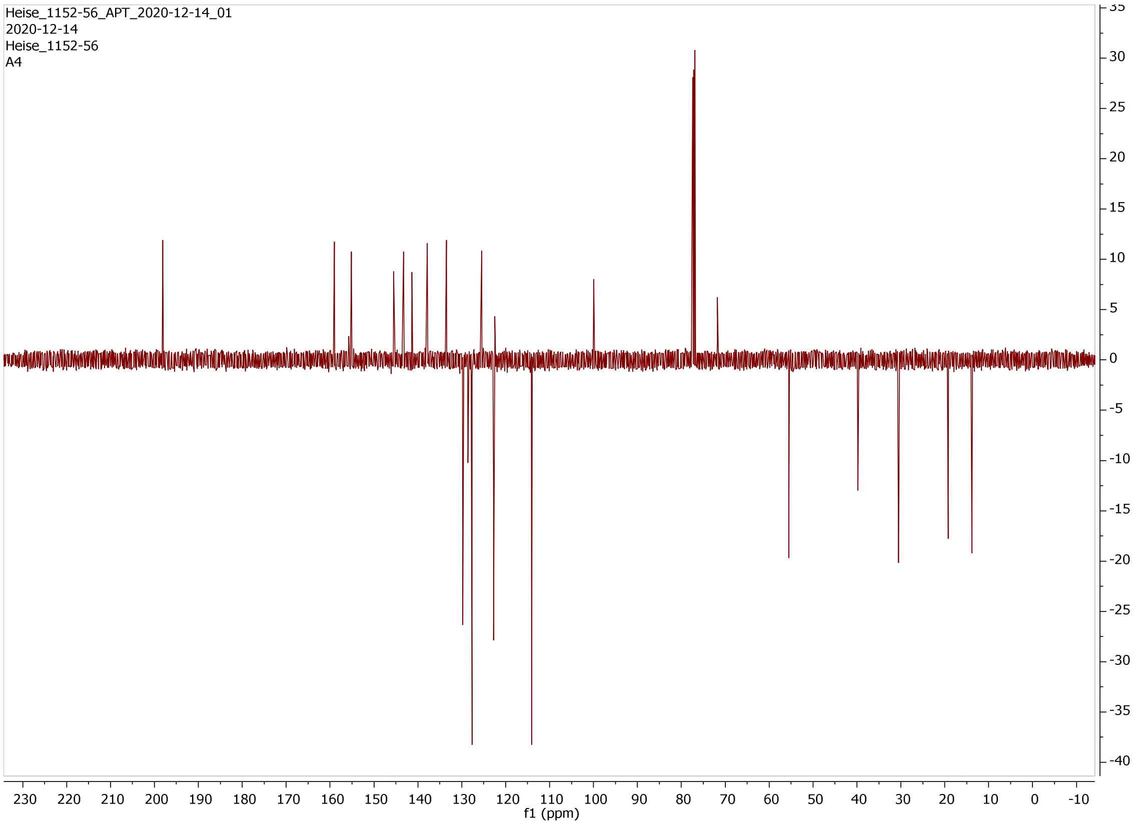

IR spectrum (ATR):

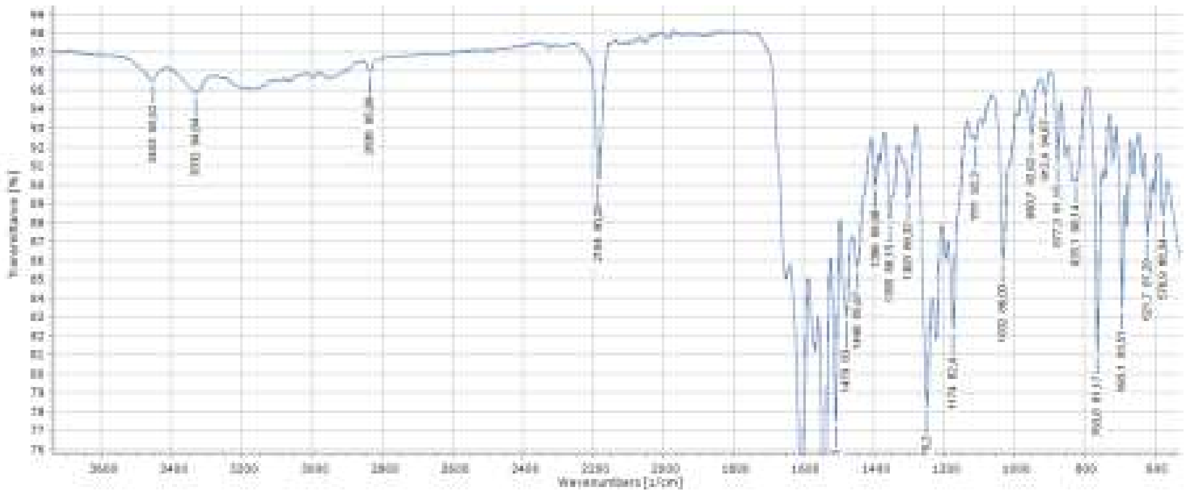

Spectra of 7e:

^{1}H NMR (500 MHz, $CDCl_3$):

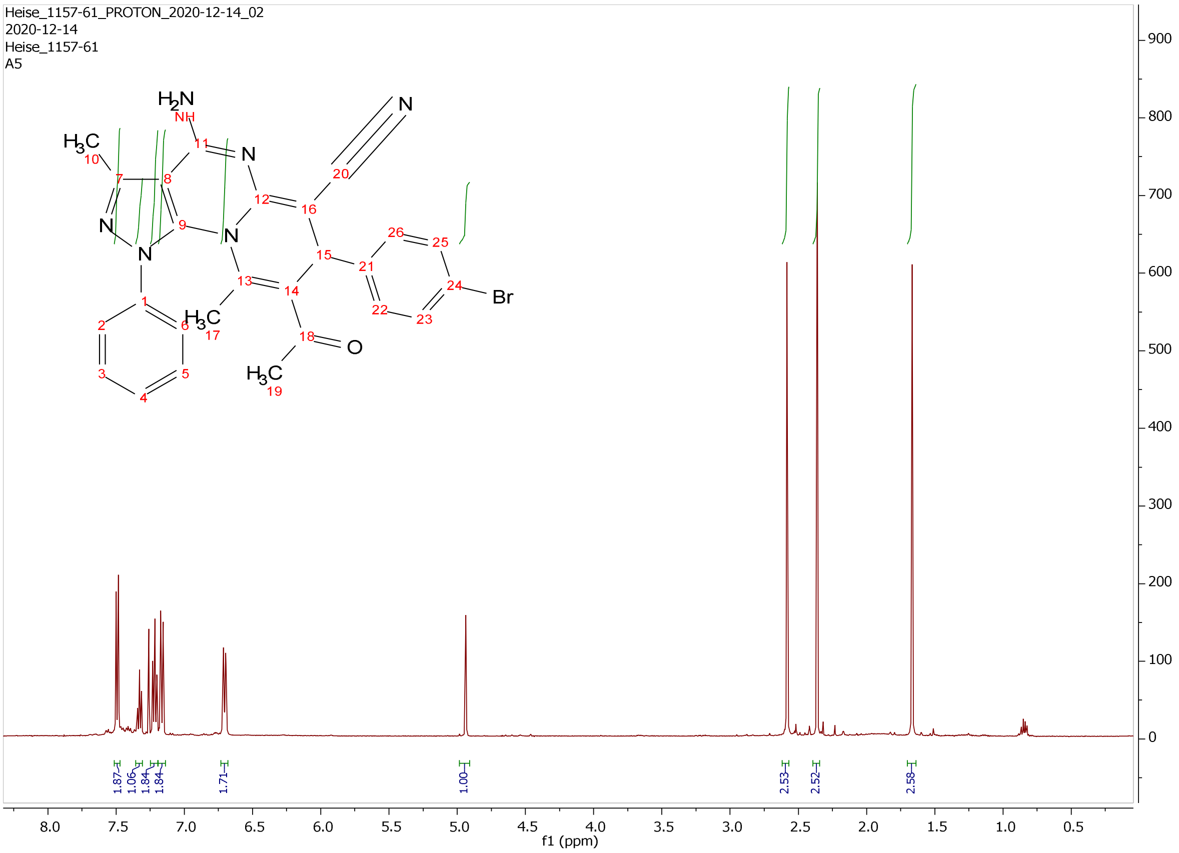

^{13}C NMR (126 MHz, $CDCl_3$):

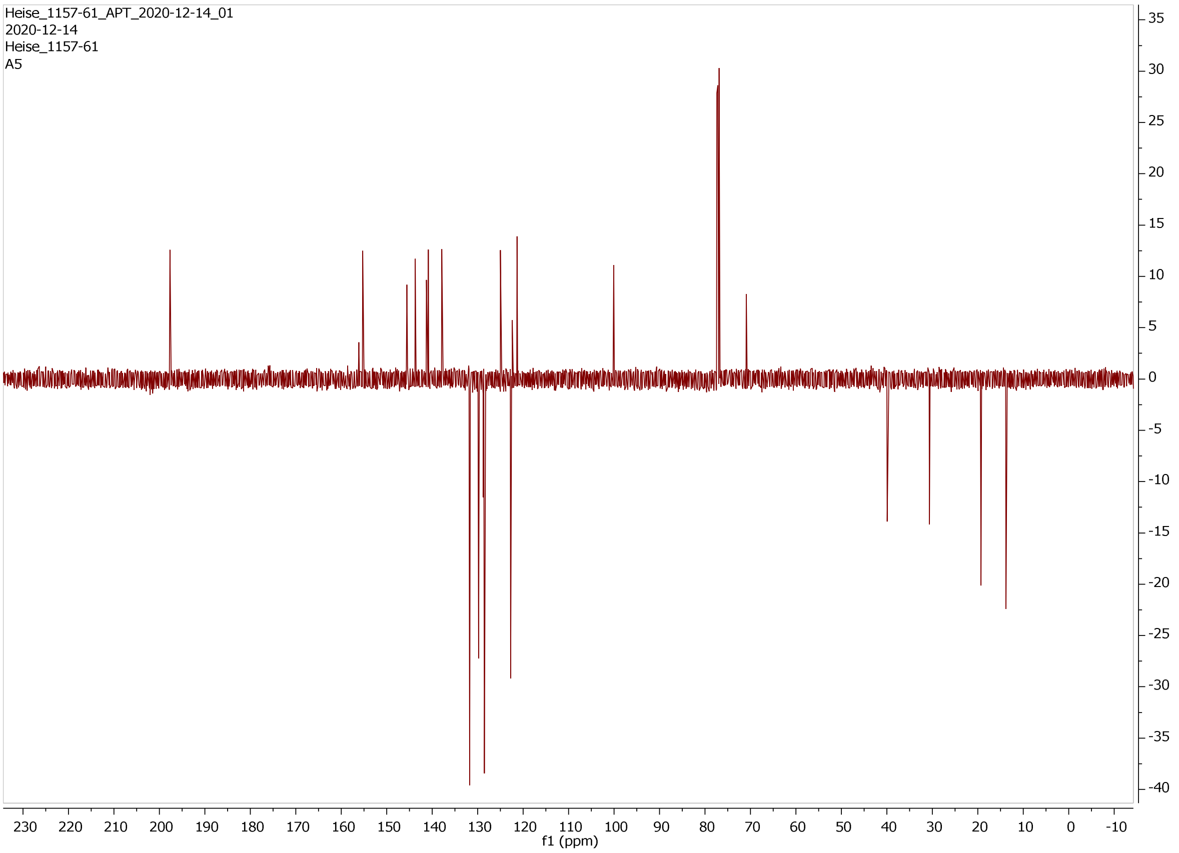

IR spectrum (ATR):

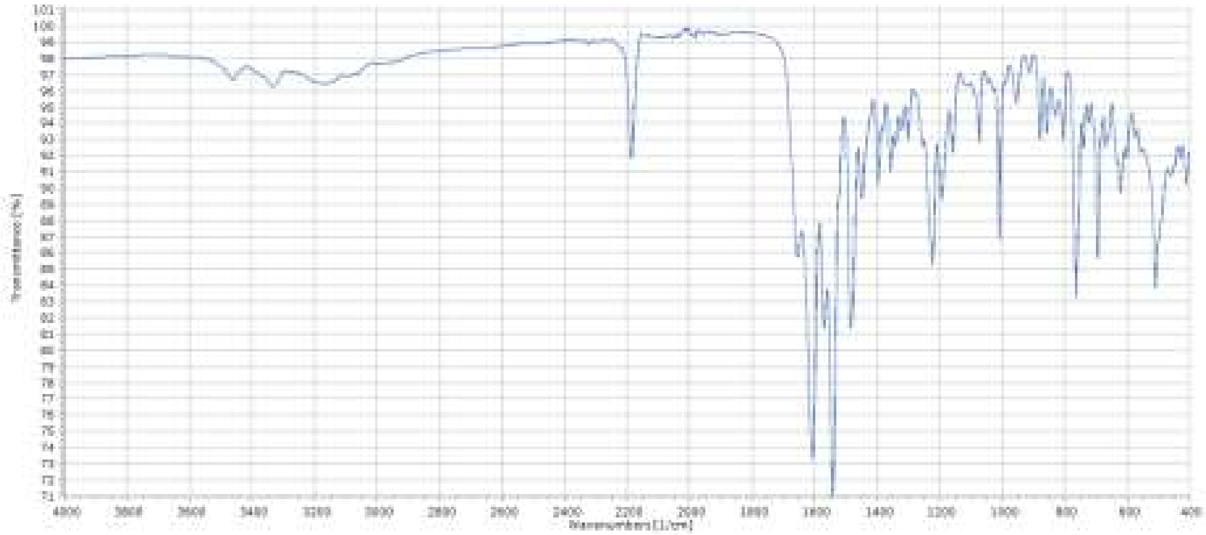

Spectra of 7f:

^{1}H NMR (500 MHz, $CDCl_3$):

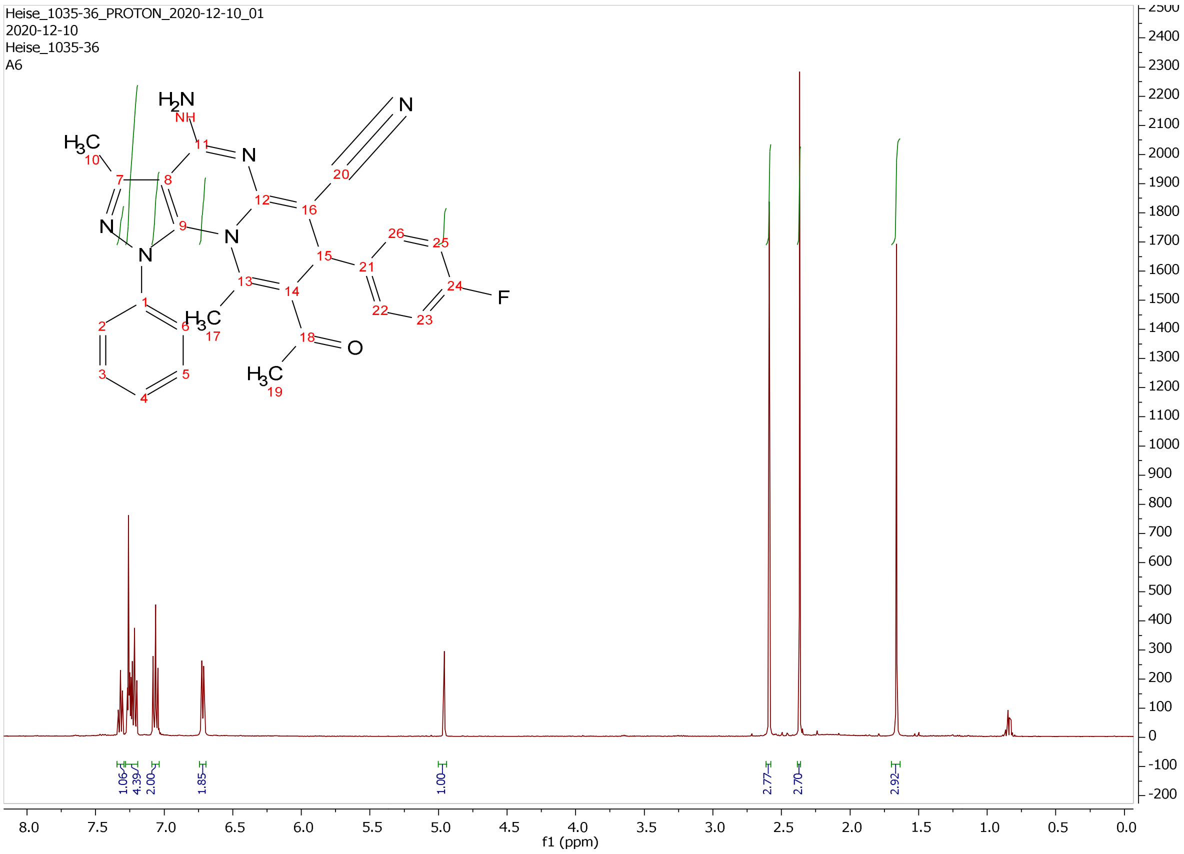

^{13}C NMR (126 MHz, $CDCl_3$):

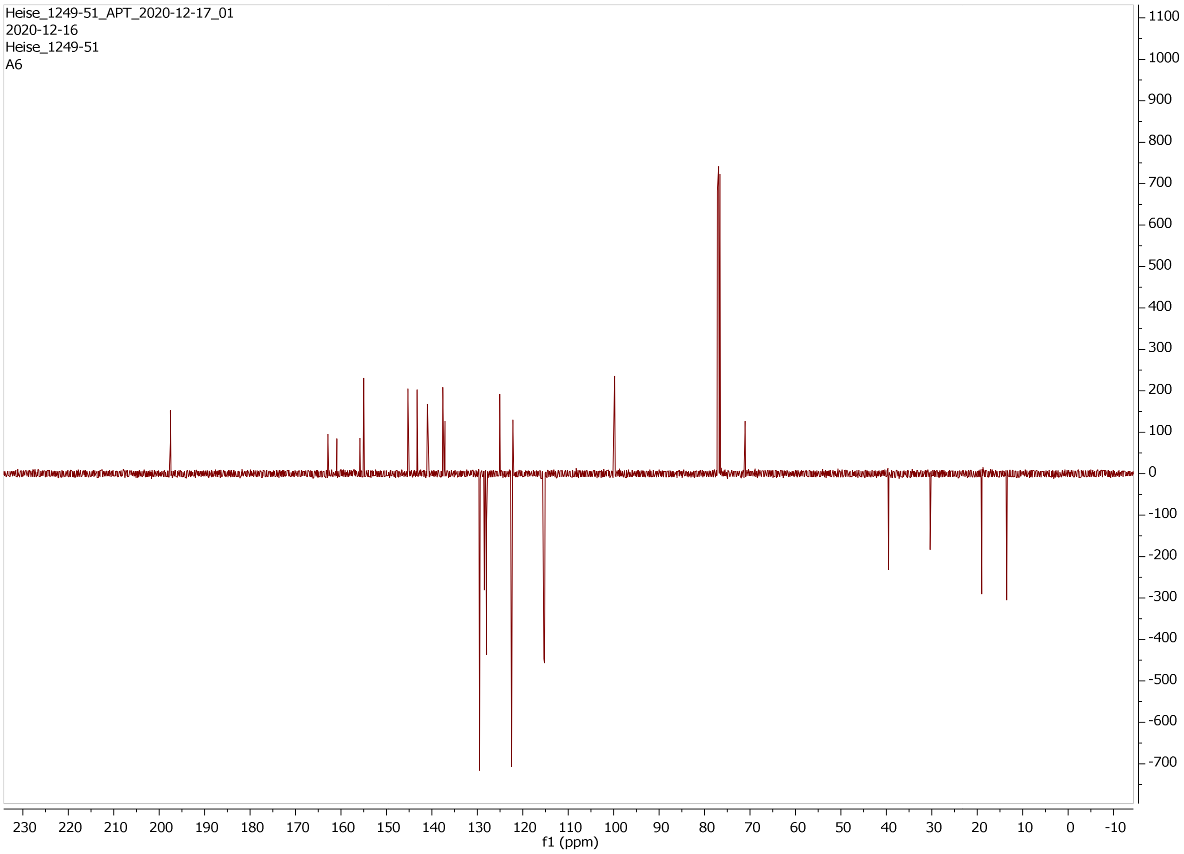

IR spectrum (ATR):

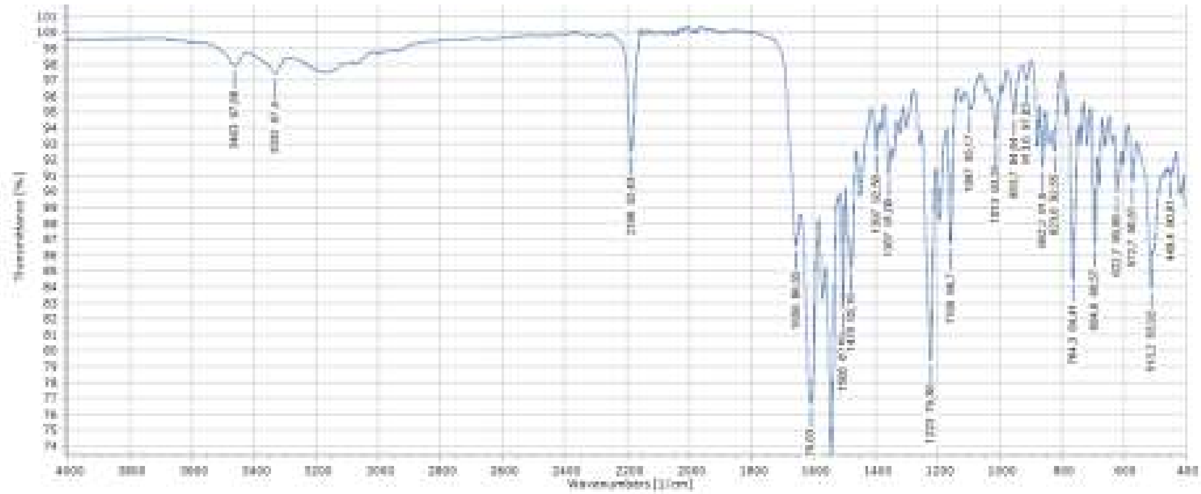

Spectra of 7g:

^{1}H NMR (500 MHz, $CDCl_3$):

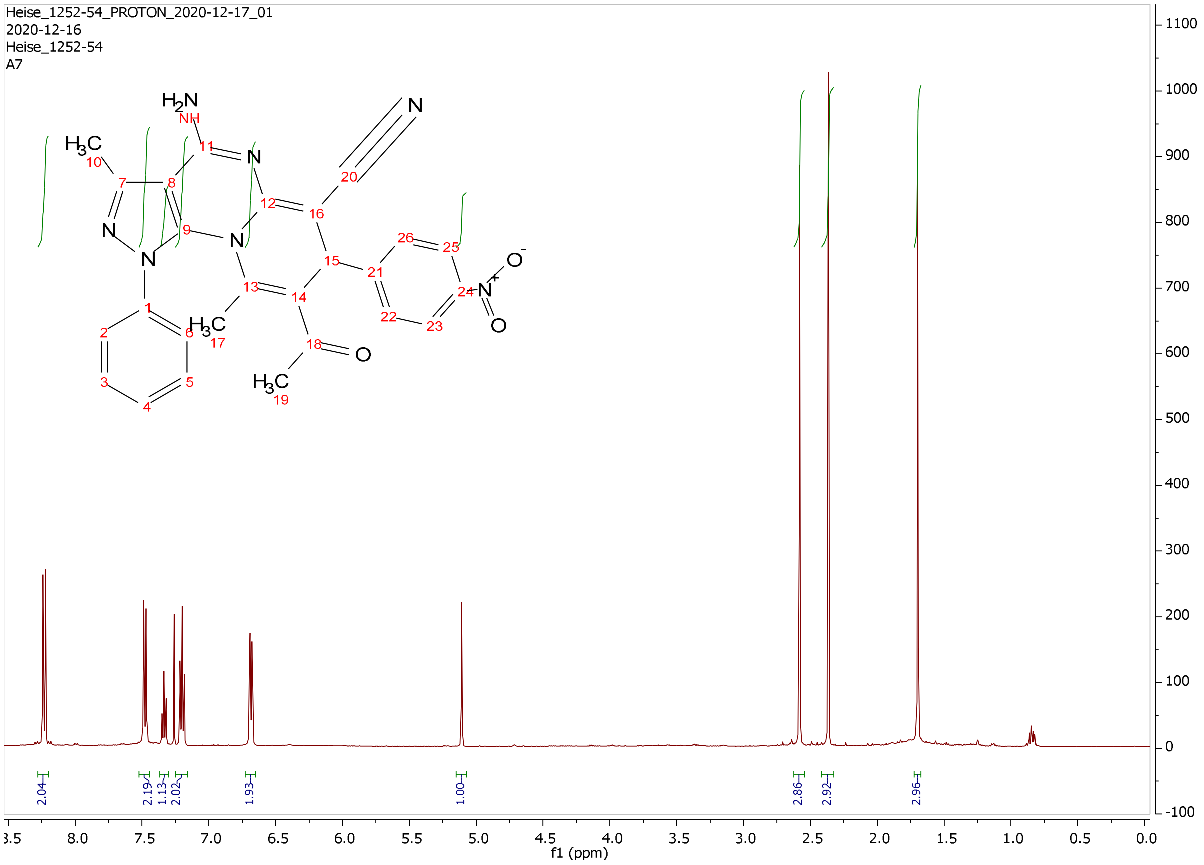

^{13}C NMR (126 MHz, $CDCl_3$):

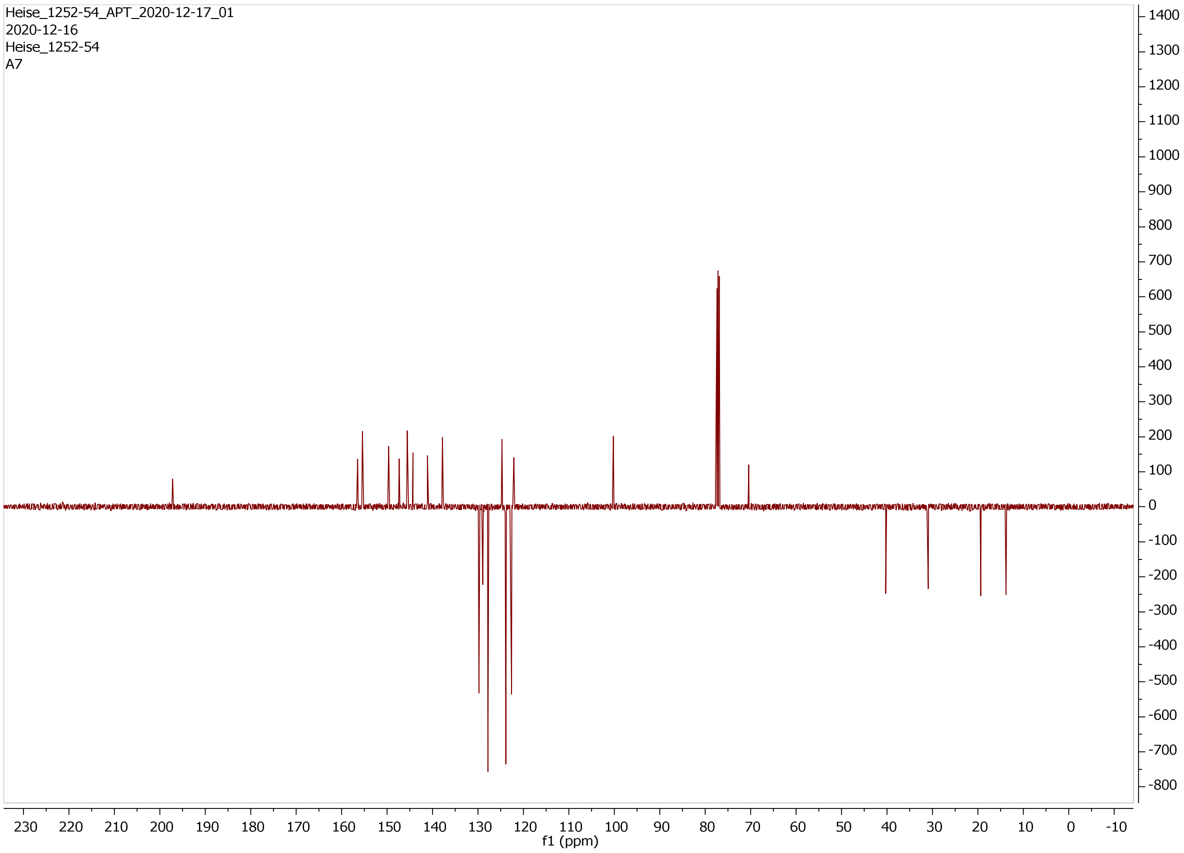

IR spectrum (ATR):

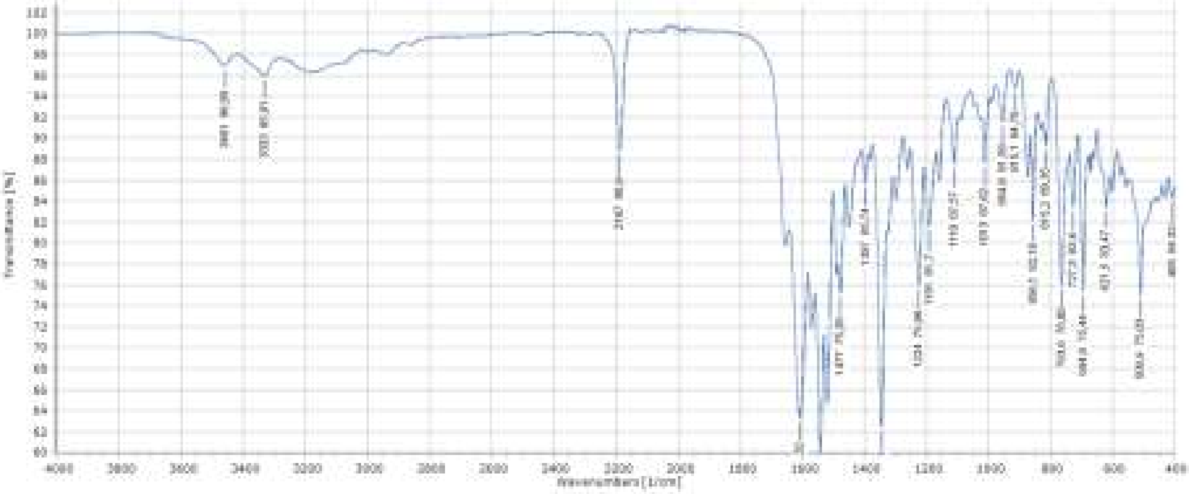

Spectra of 7h:

^{1}H NMR (500 MHz, $CDCl_3$):

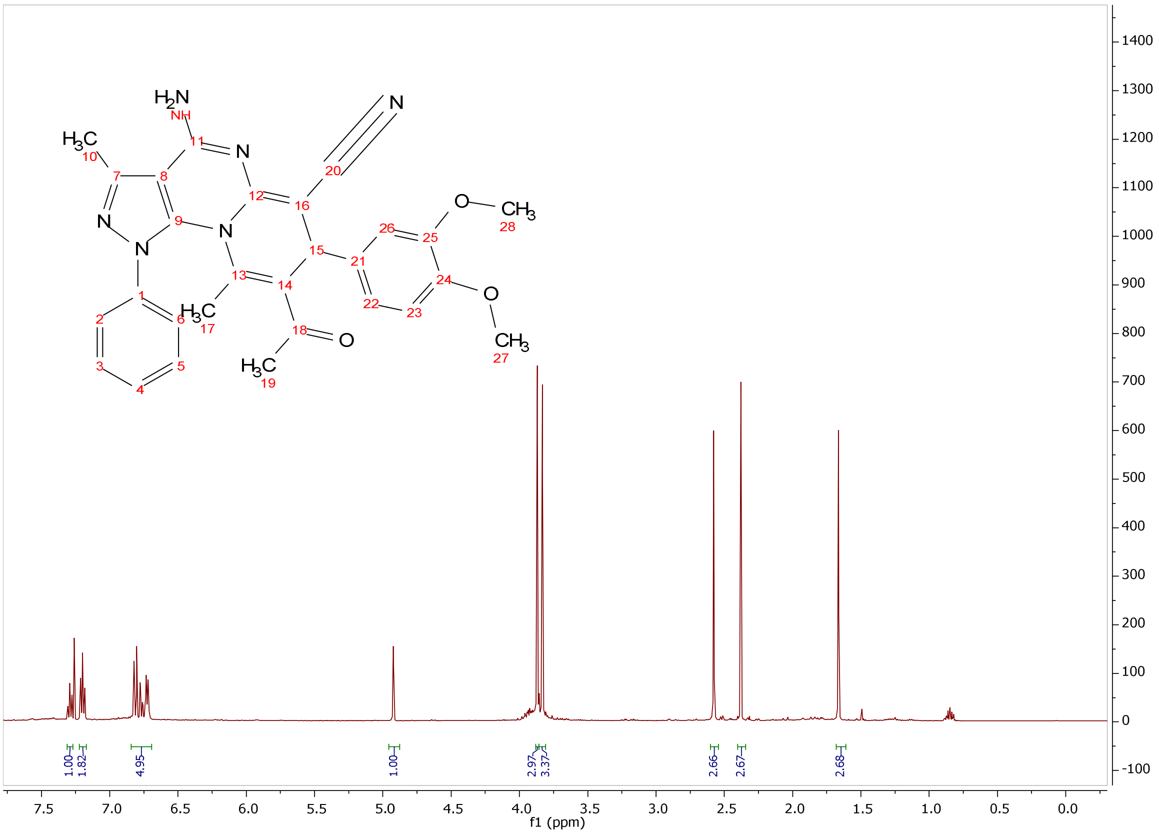

^{13}C NMR (126 MHz, $CDCl_3$):

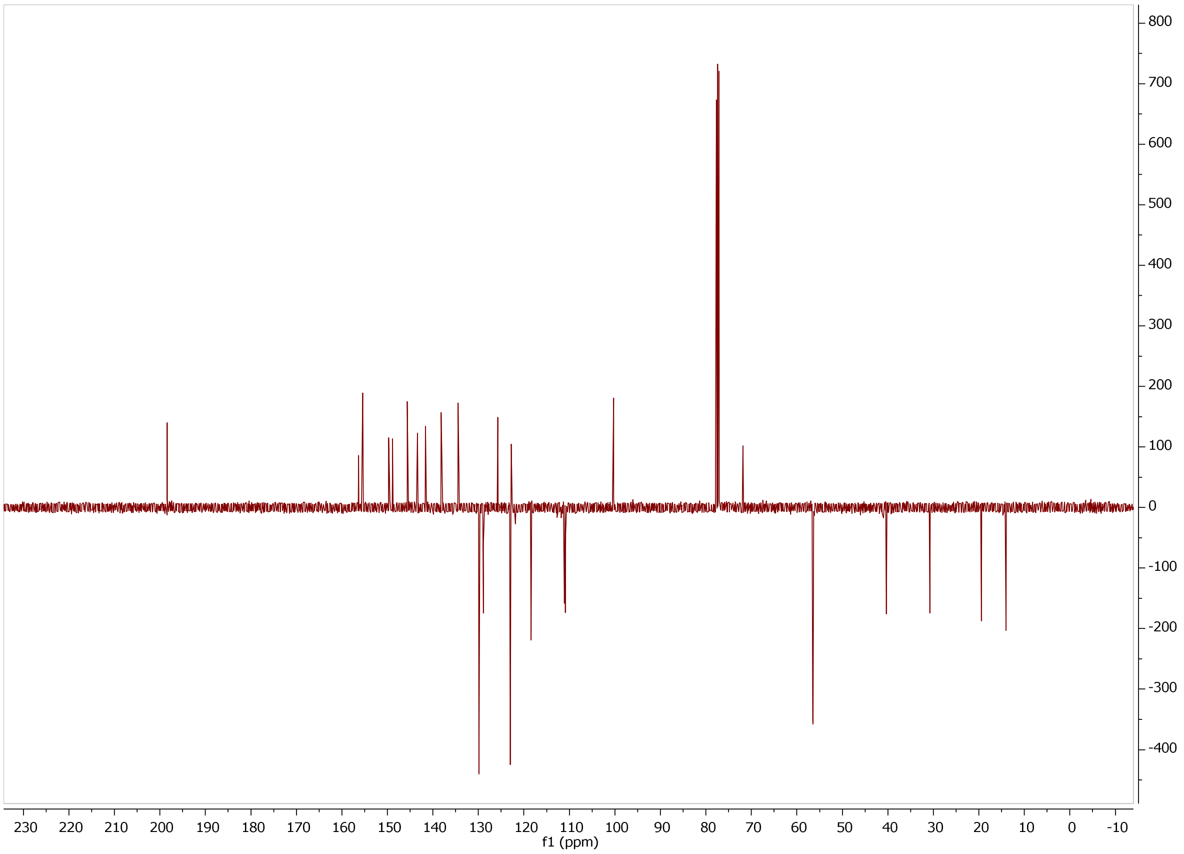

IR spectrum (ATR):

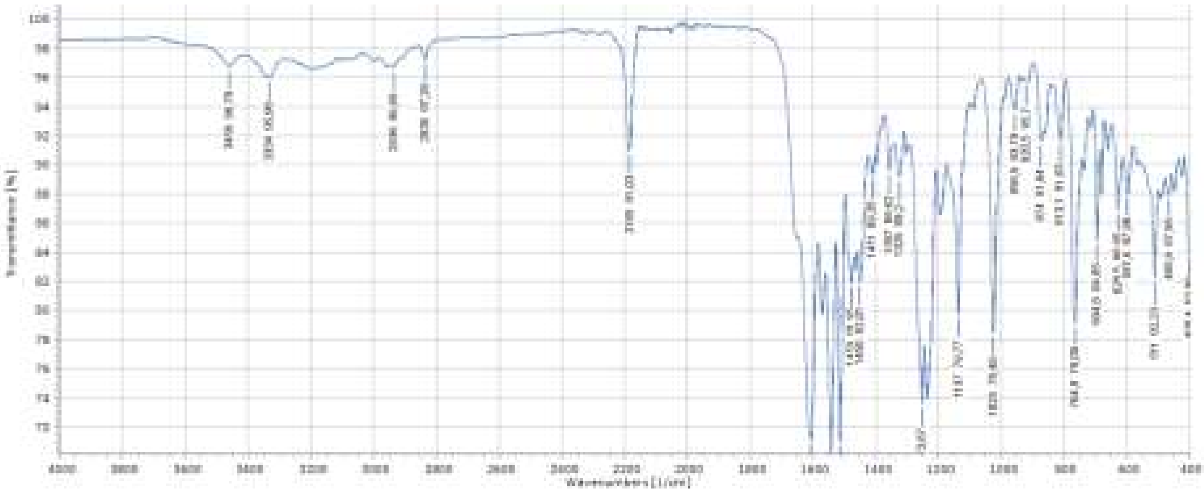

Spectra of 7i:

^{1}H NMR (500 MHz, $CDCl_3$):

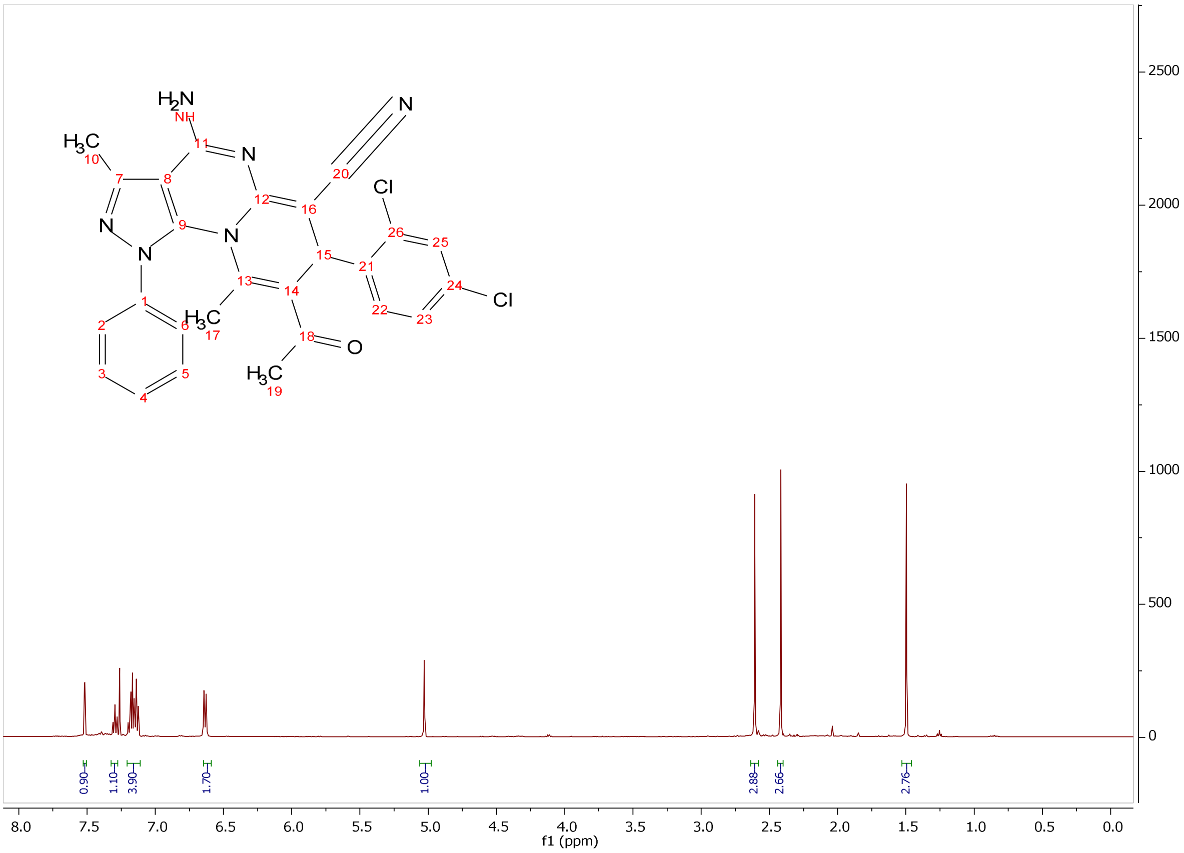

^{13}C NMR (126 MHz, $CDCl_3$):

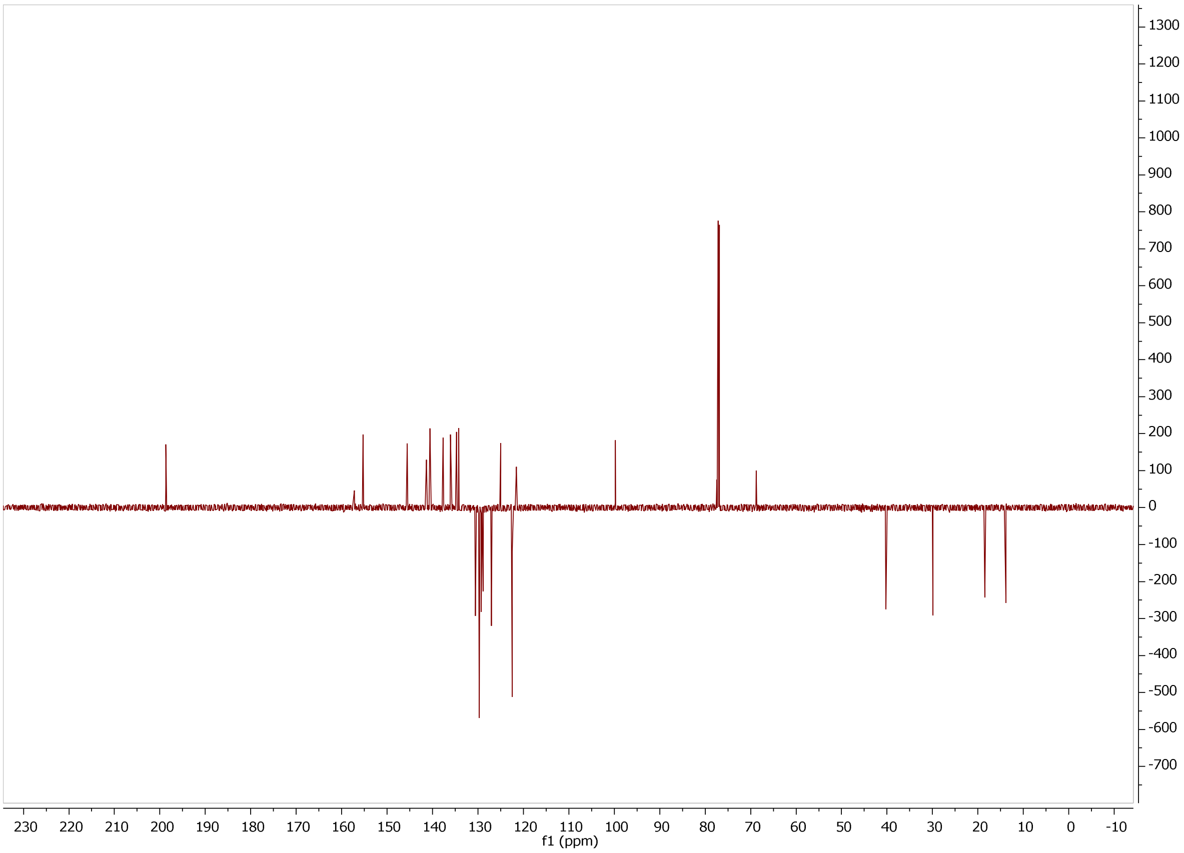

IR spectrum (ATR):

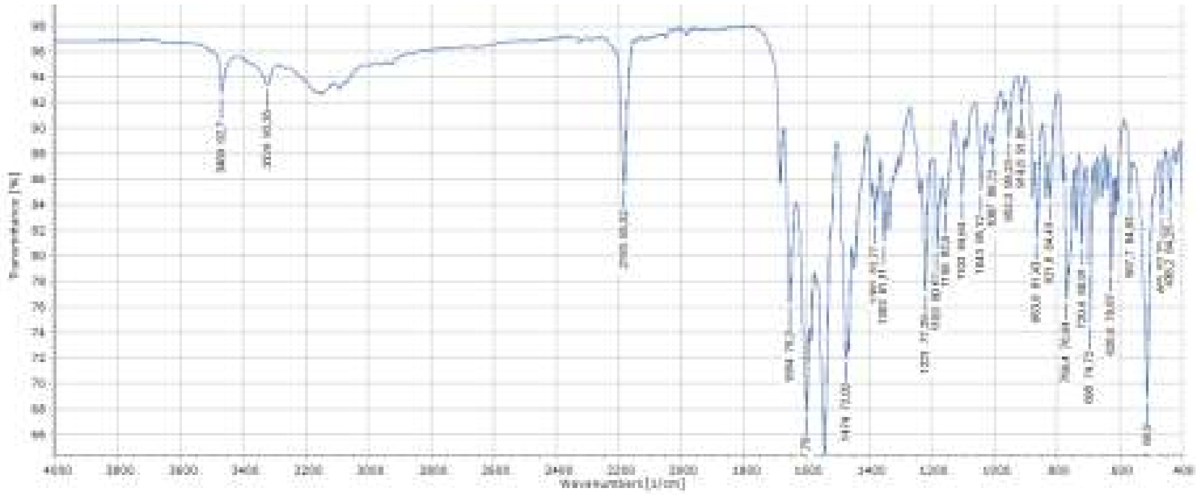

Spectra of 7j:

^1H NMR (500 MHz, $CDCl_3$):

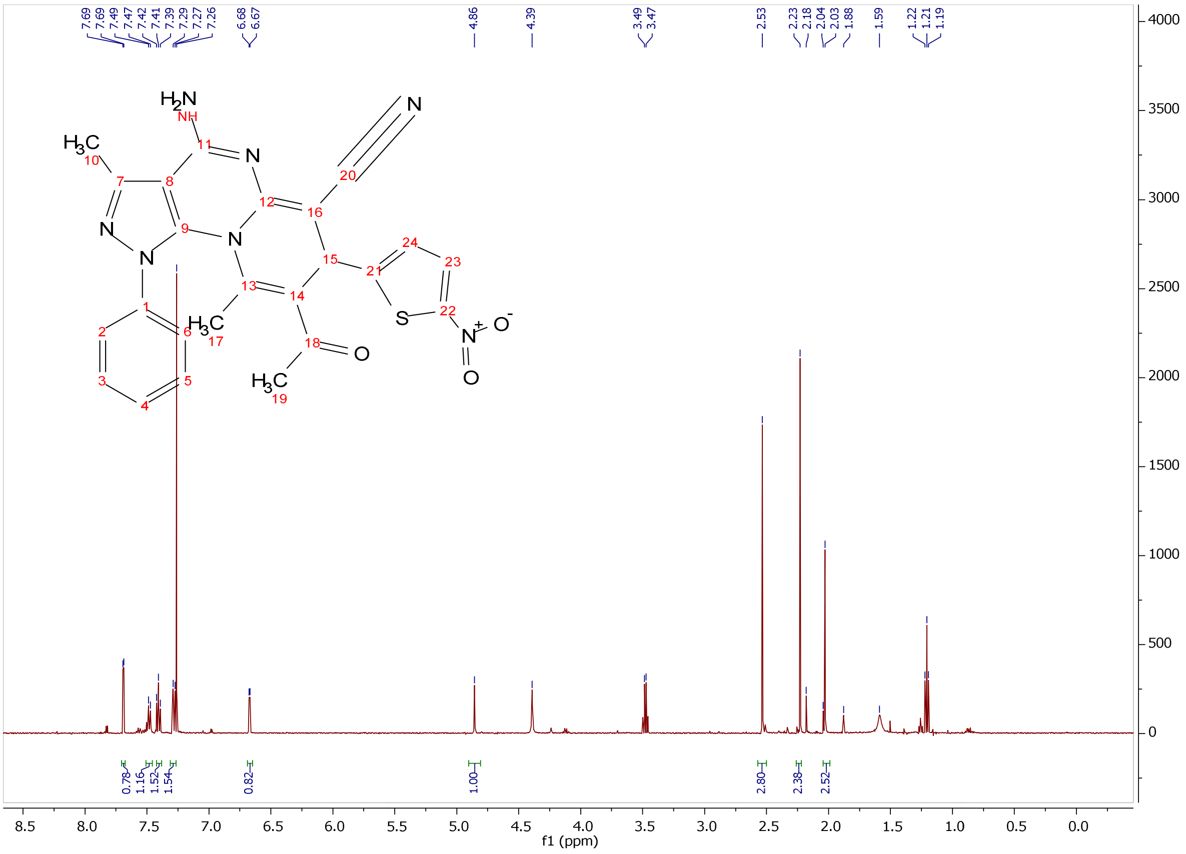

^{13}C NMR (126 MHz, $CDCl_3$):

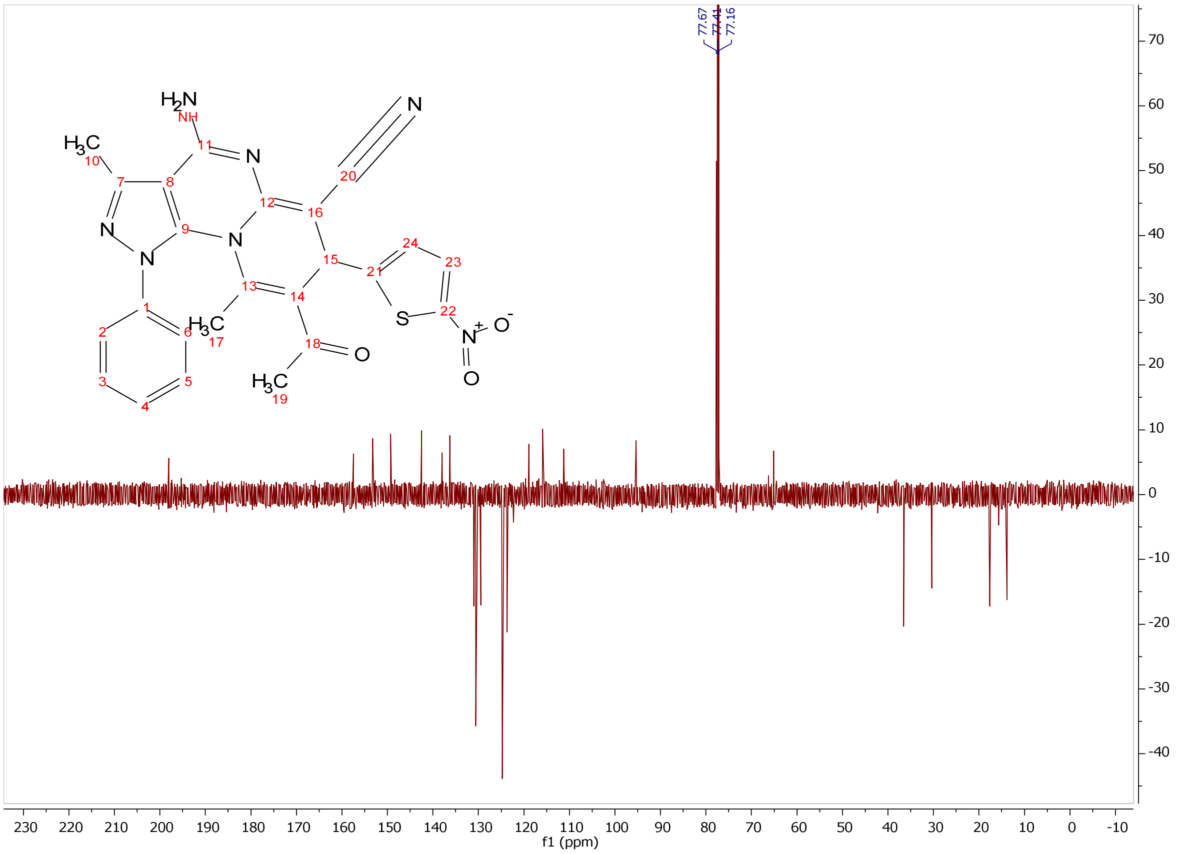

IR spectrum (ATR):

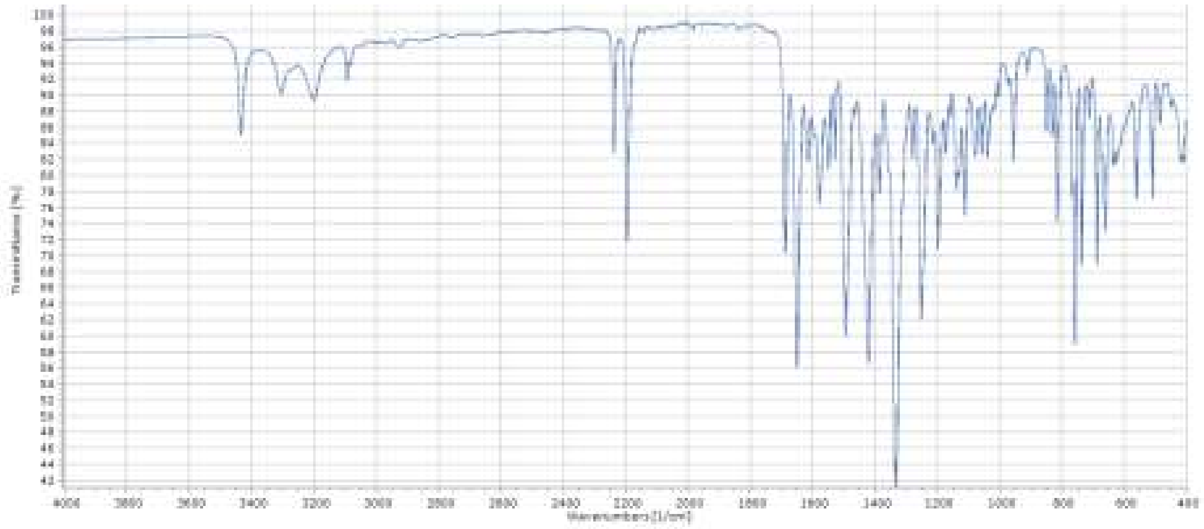

Spectra of 7k:

^1H NMR (500 MHz, $CDCl_3$):

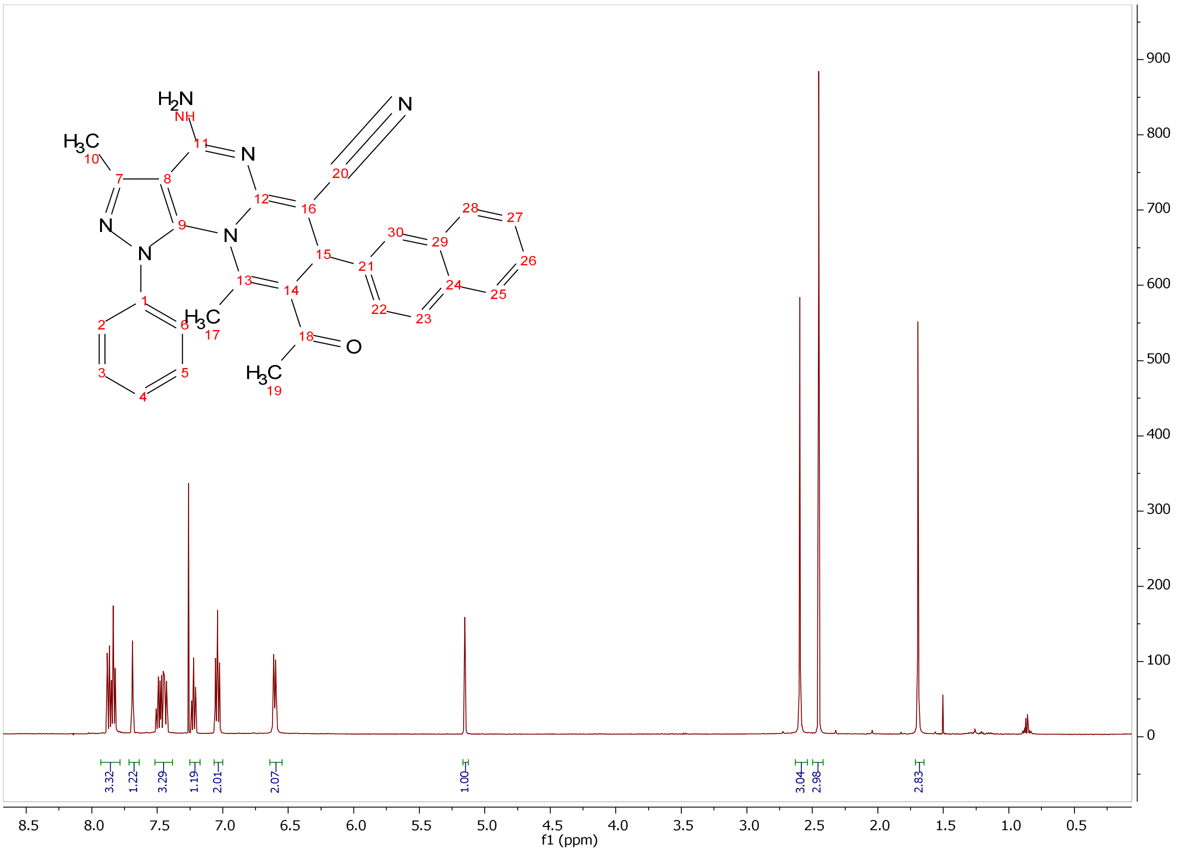

^{13}C NMR (126 MHz, $CDCl_3$):

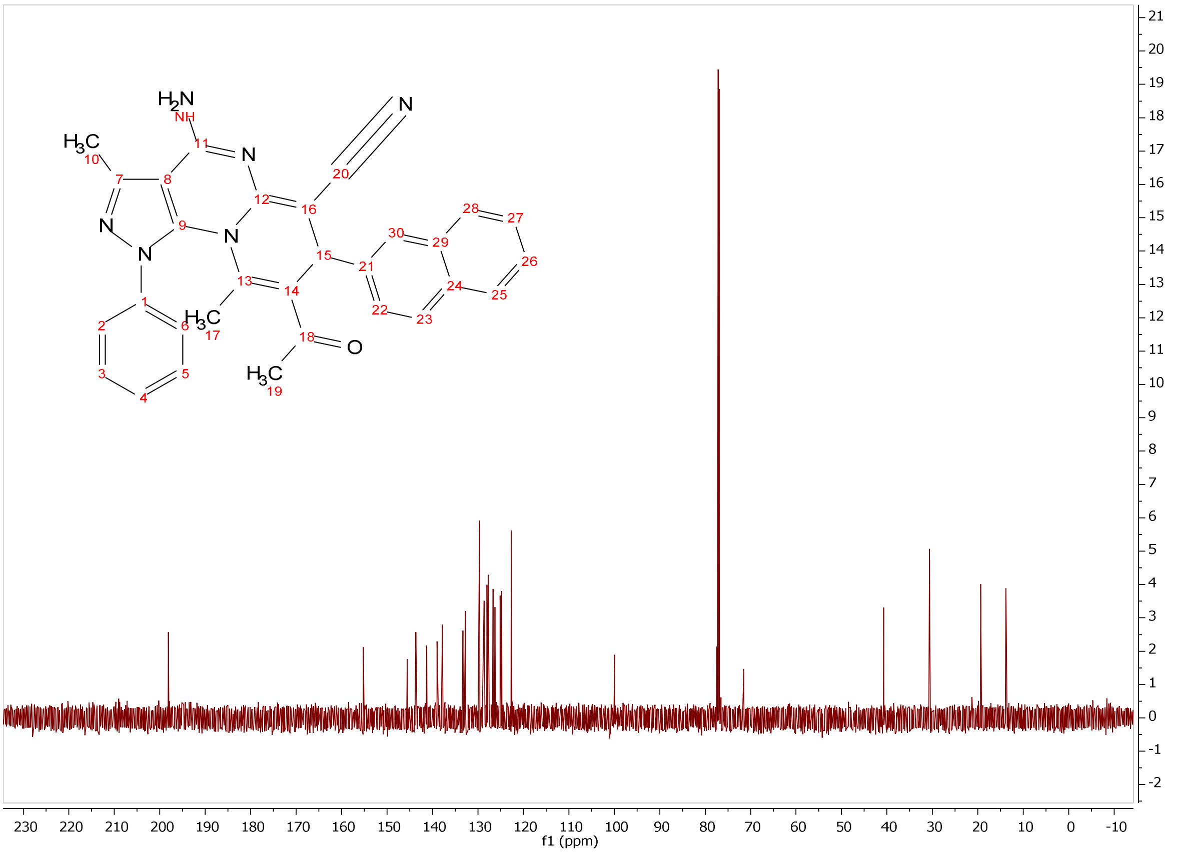

IR spectrum (ATR):

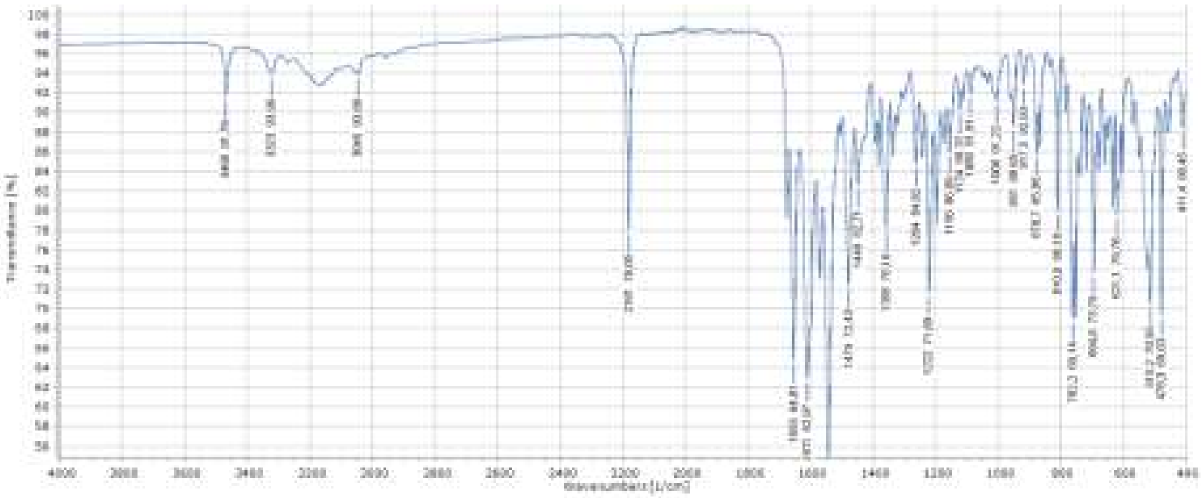

Spectra of 7l:

^{1}H NMR (500 MHz, $CDCl_3$):

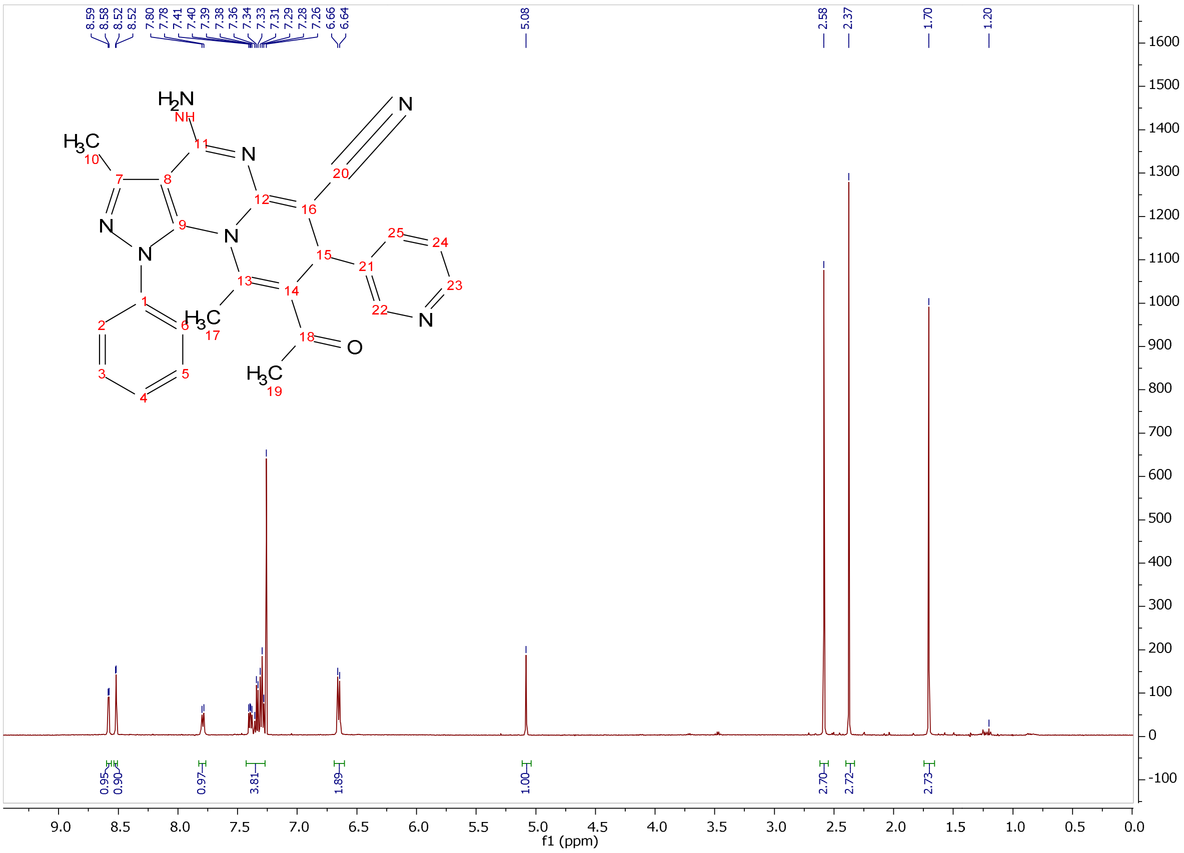

^{13}C NMR (126 MHz, $CDCl_3$):

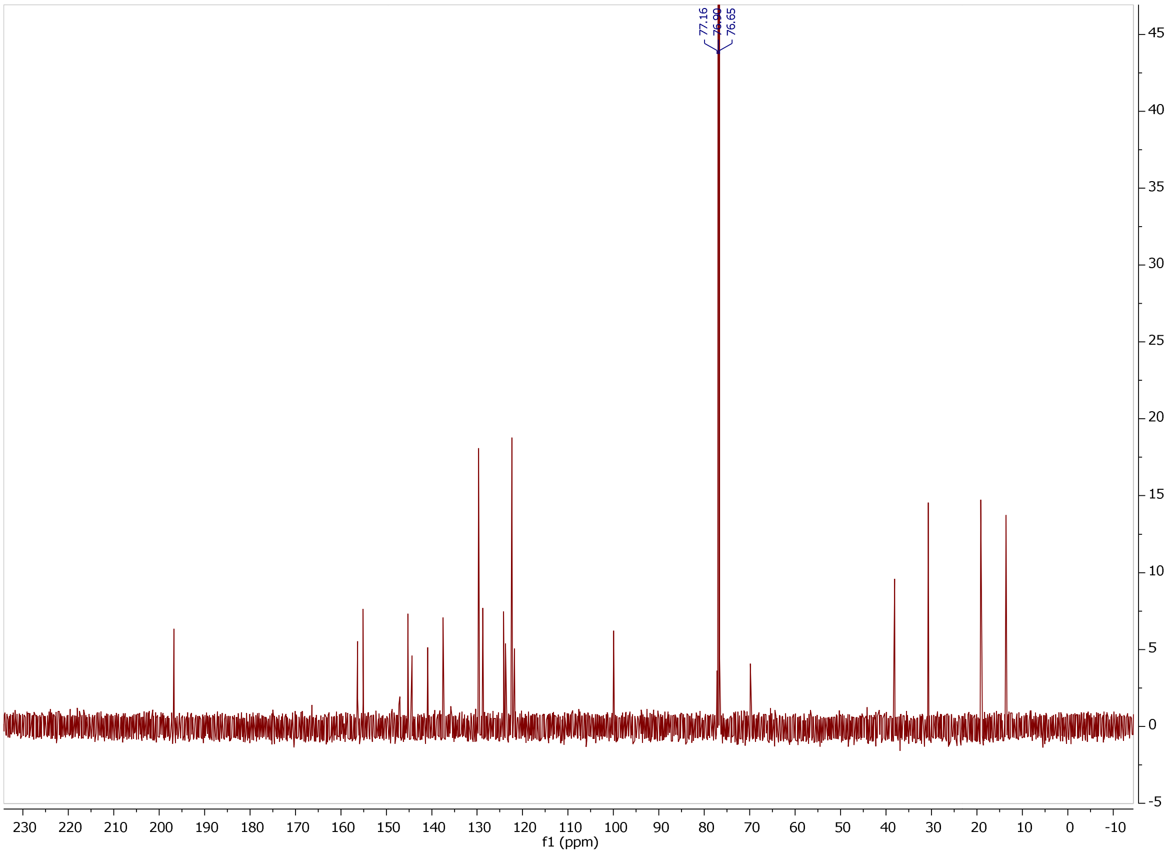

IR spectrum (ATR):

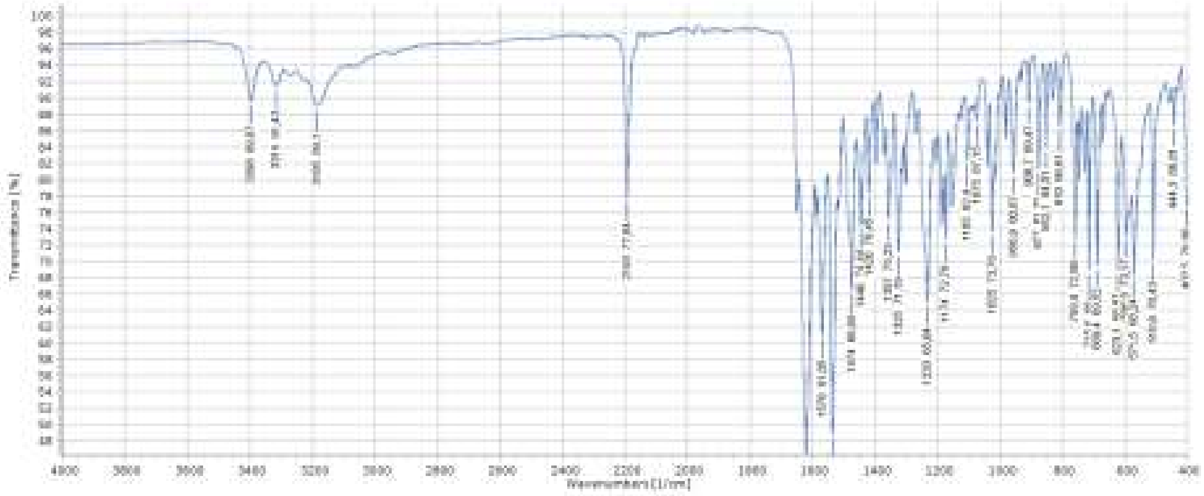

Supplement: Supplementary file 1 [file ijms-22-10258-s001.zip › ijms-1392093-supplementary.pdf]
